# Supplementary material for: Testing the efficacy of lionfish traps in the northern Gulf of Mexico
Source: PLoS One. 2020 Aug 26;15(8):e0230985. doi: 10.1371/journal.pone.0230985 (PMC7449463; doi:10.1371/journal.pone.0230985)
Supplement: S1 Data — (ZIP) [file pone.0230985.s006.zip › Final manuscript - Harris - Lionfish traps.doc]

Testing the efficacy of lionfish traps in the northern Gulf of Mexico

Holden E. Harris^1,2*^, Alexander Q. Fogg^3^, Stephen R. Gittings^4^, Robert N. M. Ahrens^2^, Micheal S. Allen^2,5^, and William F. Patterson III^2^

^1^School of Natural Resources and Environment, Institute of Food and Agriculture Sciences, University of Florida, Gainesville, Florida, United States of America

^2^Department of Fisheries and Aquatic Sciences, School of Forest Resources and Conservation, Institute of Food and Agriculture Sciences, University of Florida, Gainesville, Florida, United States of America

^3^Okaloosa County Board of County Commissioners, Destin – Fort Walton Beach, Florida, United States of America

^4^Office of National Marine Sanctuaries, National Oceanic and Atmospheric Administration, Silver Spring, Maryland, United States of America

^5^Nature Coast Biological Station, Institute of Food and Agriculture Sciences, University of Florida, Cedar Key, Florida, United States of America

*Corresponding author: Holden Earl Harris | [holdenharris@ufl.edu](mailto:holdenharris@ufl.edu) | +1-904-476-0984

# Abstract

Spearfishing is currently the primary approach for removing invasive lionfish *(Pterois volitans/miles*) to mitigate their impacts on western Atlantic marine ecosystems, but a substantial portion of lionfish spawning biomass is beyond the depth limits of SCUBA. Innovative technologies may offer a means to target deepwater populations and allow for the development of a lionfish trap fishery, but the removal efficiency and potential environmental impacts of lionfish traps have not been evaluated. We tested a collapsible non-containment trap (the ‘Gittings trap’) near artificial reefs in the northern Gulf of Mexico. A total of 327 lionfish and 28 fish that were native species (four were species protected with regulations) recruited (i.e., the number of fish observed within the trap footprint at the time of retrieval) to traps during 82 trap sets, catching 144 lionfish and 29 native species (one more than recruited indicating detection error). Lionfish recruitment was highest for single (versus paired) traps deployed <15 m from reefs with a 1-day soak time, for which mean lionfish and native species recruitment per trap were approximately 5 and 0.1, respectively. Lionfish from traps were an average of 19 mm or 78 g larger than those caught spearfishing. Community impacts from Gittings traps appeared minimal given that recruitment rates were >10X higher for lionfish than native fishes and that traps did not move on the bottom during two major storm events, although further testing will be necessary to test trap movement with surface floats. Additional research should also focus on design and operational modifications to improve Gittings trap deployment success (68% successfully opened on the seabed) and reduce lionfish escapement (56% escaped from traps upon retrieval). While removal efficiency for lionfish demonstrated by traps (12–24%) was far below that of spearfishing, Gittings traps appear suitable for future development and testing on deepwater natural reefs, which constitute >90% of the region’s reef habitat.

# Introduction

Invasive Indo-Pacific lionfish (*Pterois volitans/miles* complex, hereafter “lionfish”) are now well established in the western Atlantic, including the Caribbean Sea and Gulf of Mexico [1], and have recently invaded the Mediterranean Sea [2]. Lionfish occupy a wide diversity of invaded marine habitats, including coral reefs, subtropical artificial and natural reefs [3], seagrass beds [4], mangroves [5], estuaries [6], mesophotic reefs [7–9], and upper continental slope reefs [10]. High population densities of lionfish [3,11] have caused reductions in native reef fish abundances [12,13], altered marine communities [14,15], and likely exacerbate current stressors on marine systems [16,17]. As invasive lionfish populations do not appear to be controlled by native predators [18–20], reducing lionfish biomass is a top priority for marine resource managers [21,22]. Population and ecosystem models predict that high levels of fishing mortality over a broad geographic range will be necessary to control lionfish populations on a regional scale [15,23–25].

Lionfish are primarily removed by spearfishing on SCUBA [21,26]; however, invasive lionfish have been observed at depths >300 m [10] and diver removals are generally limited to depths <40 m. Over 557,000 km^2^ of benthic habitat in the western Atlantic invaded range of lionfish lies within mesophotic and upper-bathyal depths of 40–300 m [27,28]. Although survey capacity for deepwater reefs is relatively limited [28], lionfish density on mesophotic reefs has been documented to be higher than on corresponding shallower reefs [9,29–31]. Deepwater lionfish populations likely disrupt food webs on mesophotic reefs [7] and provide refuge for larger and more fecund individuals [32]. These protected source populations can provide larvae for sink regions [25,33] and undermine shallow-water control efforts [8,32]. Innovative harvest technologies have been proposed for deepwater lionfish removals, including modifications to existing spiny lobster traps [34], weaponized remotely operated vehicles [22,35], and novel trap designs [36]. Such technologies may offer a safe method for lionfish removal from deepwater reefs inaccessible to spearfishers and could allow a single vessel to multiply effort via simultaneous gear deployments.

Collaborative work by members of non-profit organizations, Florida Fish and Wildlife Conservation Commission, and US National Oceanic and Atmospheric Administration have resulted in lionfish trap prototypes [37], which have been further developed into a non-containment trap model [38]. The collapsible non-containment ‘Gittings trap’ is designed to allow lionfish and other marine species to freely move over the trap’s footprint, with traps closing during retrieval (**Table 1, Fig 1**). Gittings traps are made from common and inexpensive materials, allowing for construction in remote locations where specialized materials may be difficult to source. A low cost of production could expand harvesting capacity for the nascent lionfish commercial fishery, which is currently constrained by inconsistent supply [39,40]. Such a commercial deepwater lionfish fishery may offer alternative livelihood strategies for fishers and improve coastal food security [41]. However, it is necessary to further evaluate a potential new harvest gear for possible undesirable effects – including bycatch, habitat damage, entanglement, and ghost fishing – before it is permitted and widely implemented. Traditional fish traps have a broad catch composition [42] and their widespread use has contributed to severe overfishing on many Caribbean coral reef systems [42,43]. Given the potential for bycatch and overfishing, moratoriums on fish traps have been in place in US Atlantic and Gulf of Mexico waters for decades [44], with the exception of a limited trap fishery for Atlantic black sea bass (*Centropristis striata*) [45].

**Table 1. Gittings trap characteristics.** Goals of the Gittings trap with design attributes employed to achieve those goals.

| **Goals** | | **Trap characteristics** |
| --- | --- | --- |
| Attract and capture lionfish | Plastic lattice provides vertical structure to attract lionfish from neighboring habitats. | |
| Avoid bycatch of native species | Non-containment design prevents fish mortality prior to retrieval. Lack of bait reduces recruitment of non-targeted species. Presence of lionfish may also deter native species. | |
| Prevent ghost fishing if lost | Non-containment design with downward-opening curtain minimizes the likelihood of ghost fishing if a trap is lost. | |
| Prevent habitat damage | Collapsed trap falls quickly through the water to facilitate placement accuracy. Traps are placed on sand and low relief habitats where snagging is less likely. Low center of gravity reduces likelihood of movement. | |
| Allow for easy transport on fishing boats | Traps are collapsible and stackable. | |
| Allow for safe release of bycatch | Able to recompress bycatch by descending in a closed trap, releasing fish upon contact with the bottom. | |

**Fig 1. Schematic of Gittings lionfish trap deployment.** Traps are designed to A) descend closed and B) open when the curved deflectors contact the seafloor. C) The traps remain open during deployment then close when the trap is ascended during retrieval.

Here, we report results from testing Gittings traps near artificial reefs in the northern Gulf of Mexico (nGOM). We examined gear performance and how lionfish and native species recruitment (number of fish observed within the trap footprint at the time of retrieval) and catches (fish landed aboard the vessel from a trap) were affected by the lionfish density on the adjacent artificial reef and different Gittings trap deployment configurations, including soak time, the number of Gittings traps deployed, and their distance from the adjacent artificial reef. Lionfish size distributions was compared between Gittings trap catches by distance to the artificial reef and *in situ* size distributions obtained from spearfishing catches. Gear testing Gittings trap performance involved evaluating deployment success (% of traps that successfully opened on the seabed), lionfish escapement (% of individuals that escaped traps upon retrieval), and whether traps moved on the seabed while deployed. We consider how findings from this initial study can inform further research and development of lionfish traps and innovative harvest technologies to control deepwater lionfish populations.

# Methods

Twelve Gittings traps were constructed in May and June 2018. Gittings traps have hinged jaws that allow for the trap to remain closed and travel vertically through , water column (**Fig 1A**) then open when the curved deflectors contact the seafloor the bottom (**Fig 1B**). Trap jaws were made from 4.5 m long sections of #6 rebar (19 mm diameter) bent into two half-hoops with a curved extension on one end of each jaw to act as deflectors for opening the trap **(Fig. 2)**. The jaws pivot around a 2 m long center axle made with #6 round bar. The axle and jaws are connected with two metal hinge plates (4 cm x 10 cm) each with holes approximately 20 mm in diameter. Trap netting consisted of 3 m^2^ of 22 mm diameter mesh nylon netting (#420 green knotless). A sheet of plastic lattice (71 cm x 75 cm with 2.5 cm openings) provided vertical structure for attracting lionfish (**Fig. 1C, Fig 2**). A two-line harness was attached to the apex of each trap jaw using 12-strand Dyneema fiber rope (Amsteel Blue, 2.78 mm diameter). To prevent this line from fouling within the trap, an inline syntactic foam float was secured at the apex of the harness. An instructional video for building similar Gittings traps is provided at <https://youtu.be/ta8WInxyXFA>.

**Fig 2. In water photos of a Gittings lionfish traps deployed near artificial reefs in the northern Gulf of Mexico.** Lionfish are attracted to the structure made by plastic lattice. The trap jaws are constructed from rebar and bent to make deflectors that open the trap when it contacts the seafloor. The jaws open around a central pivot axle connected with a hinge plate. Fish are captured in the mesh nylon netting when the jaws are lifted via a two-line harness. Images: A. Fogg and H. Harris.

Gittings traps were deployed in depths of 33–37 m near eight artificial reefs on the nGOM Florida shelf. These included four poultry transport units (i.e., chicken coops), one steel pyramid, one cement mixer, and two military tanks located approximately 30 km south of Destin, Florida (**Fig. 3**). The artificial reefs were approximately 3 m x 2 m x 2 m (length x width x height) in size and deployed on sand bottom. No other known reef habitats (artificial or natural) were within at least 300 m of a given artificial reef. Lionfish density at each reef was surveyed immediately prior to trap deployment and trap retrieval. Surveys were conducted with point-counts by divers on SCUBA within a 15-m wide cylinder with the artificial reef at the center [46]. The diver survey included a lionfish count on the opposite sides of the artificial reef, followed by a count of lionfish within the reef structure [46,47]. Lionfish density (fish per 100 m^2^) was computed as abundance (number counted) divided by the area sampled (177 m^2^).

**Fig 3. Study site locations for testing Gittings lionfish traps.** A) Traps were deployed adjacent to artificial reefs approximately 30 km offshore NW Florida in depths of 33–37 m. The eight reef study sites were B) separated by >300 m and C) located in the northern Gulf of Mexico.

Trap deployments and retrievals (n = 58 replicates; **Table 2**) were conducted during June through December 2018. Deployment factors for number of traps (two levels: single or paired), distance to reef [three levels: near (5 m), intermediate (15 m), or far (65 m)], and soak time (categorical with five levels: 0.25 d, 1 d, 4–5 d, 8 d, or 12–14 d) were randomized for each deployment. Traps were deployed from the vessel and allowed to descend freely. Single-trap treatments included one trap deployed at the reef. Paired-trap treatments included two traps deployed simultaneously ~3 m apart. Deployment success (i.e., a trap landed upright and opened) for each trap deployment (n = 82 total traps deployed including paired treatments) was noted by SCUBA divers during the survey and unsuccessful deployments were corrected. Underwater visibility ranged from 5–12 m, thus near traps were within direct visual range of the reefs by divers, intermediate distanced traps were sometimes within visual range or just outside, and far traps were outside of visual range. Traps were retrieved by SCUBA divers using two, 22-kg lift bags filled with air (video of trap retrieval provided at <https://youtu.be/Tf8K6ZwQV_Y>). Recruitment of lionfish and native species to the footprint of the trap was documented by a SCUBA diver prior to retrieval and subsequently compared to the catch. Although we attempted to use time-lapse camera units to document higher resolution recruitment to the traps, the camera failure rates during this study were too high for the data to be useful.

**Table 2. Replicates by deployment factor tested.** Number of Gittings trap replicates by deployment configuration with factors of soak time (0.25, 1, 4-5, 8, or 12-14 days), number of traps deployed (single or paired), and distance to artificial reef (5, 15, or 65 m).

| **Soak time /** | **Distance to reef** | | |
| --- | --- | --- | --- |
| **# traps** | **5 m** | **15 m** | **65 m** |
| **0.25 days** |  |  |  |
| Single | 5 | 4 | 0 |
| Paired | 0 | 0 | 0 |
| **01 day** |  |  |  |
| Single | 4 | 4 | 0 |
| Paired | 0 | 0 | 0 |
| **04-05 days** |  |  |  |
| Single | 5 | 4 | 1 |
| Paired | 3 | 3 | 1 |
| **08 days** |  |  |  |
| Single | 2 | 0 | 2 |
| Paired | 2 | 0 | 2 |
| **12-14 days** |  |  |  |
| Single | 4 | 2 | 2 |
| Paired | 4 | 2 | 2 |

Generalized linear mixed models (GLMMs) were computed to test the effect of deployment factors (i.e., number of traps, distance to artificial reef, and soak time) on 1) lionfish recruitment, 2) lionfish catch, 3) native reef fish recruitment, and 4) native reef fish (**Eq. 1**). Lionfish density on the adjacent artificial reef site was included as a covariate. Individual artificial reef sites were sampled multiple times with different deployment configurations and soak times, thus the reef site was included in the GLMMs as a random effect (random intercept) and assumed to be normally distributed with a mean of zero and variance σ^2^. Deployment factors and the lionfish density covariate were evaluated at an experiment-wise error rate (α) of 0.05. Quantile-quantile (QQ) plots were used to determine if errors were best fit with a normal, lognormal, Poisson, or negative binomial distribution. Likelihood was estimated with Laplace approximation based on GLMM fitting and inference protocols [48]. Analyses were conducted in R (version 3.6.1) using the LME4 [49] and MASS [50] packages. See supplemental material for R code and raw data. The QQ plots can be produced by running the R code.

| Recruitment_Lionfish_ ~ Negative binomial (μ) | Eq. 1 |
| --- | --- |
| Catch_Lionfish_ ~ Negative binomial (μ) |  |
| Recruitment_Native species_ ~ Negative binomial (μ) |  |
| Catch_Native species_ ~ Negative binomial (μ) |  |
| Reef ~ N(0, σ^2^) |  |
| log(μ) = *Trap number* + *Distance* + *Soak time* + *Lionfish density* + (1 \| *Reef site*) |  |

Total length (TL) was measured for trap-caught lionfish (n = 163) to 1) compare size of lionfish caught in the trap and distance from reef and 2) compare sizes of trap-caught lionfish to those caught via spearfishing (n = 3,063) at other artificial reefs in the study region. Lionfish size samples via spearfishing were collected during monthly lionfish culls by trained volunteer divers [51]. Spearfishing collections were subset for lionfish captured from artificial reefs during the same time period as the trap testing (June–December 2018). During spearfishing, divers attempted to capture all lionfish observed on a given artificial reef regardless of fish size. Lionfish detectability was assumed to be unbiased for larger individuals given the low structural complexity of the artificial reefs and the fact that previous serial removals on similar habitats produced homogeneous size distributions [46]. Nonparametric two-sample Kolmogorov–Smirnov (KS) tests were used to compare TL distributions by distance (5 m, 15 m, and 65 m) and TL distributions between trap-caught or spear-caught lionfish. KS tests were used given TL distributions were non-normal with multiple modes present. The TL data met the assumptions of the KS test, i.e., data were independent, ordinal, uncensored, ungrouped, and followed a continuous distribution. Differences in fish mean weight were estimated with allometric parameters estimated for nGOM lionfish on artificial reefs [55].

# Results

## Gear testing

Traps deployed upright and opened during 56 of 82 (68%) deployments (video of Gittings trap opening during deployment and provided at <https://youtu.be/XlyNuLxEqgQ>). Traps landed on sand and there were no entanglement issues with the habitat. The potential for Gittings trap movement was tested during two severe weather events. On Sept 4–5, 2018 the center of Tropical Storm Gordon passed ~150 km west of 12 deployed traps with maximum sustained winds of >110 km/h and recorded seas >5 m. Traps were retrieved two days later with all 12 found upright and no change in location, although traps were heavily fouled with algae (video of trap retrieval after Tropical Storm Gordon is provided at <https://youtu.be/7wZpe5fOozs>). Then, on Oct 9–10, 2018 Category 5 Hurricane Michael passed ~100 km east of six deployed traps with maximum sustained winds >250 km/h and seas >15 m. Traps were unable to be retrieved for over a month due to extensive damage in the region but, upon retrieval, all six traps were upright at their deployment locations. While these observations indicate high-energy storm events did not move Gittings traps on the seafloor, it is currently unclear if or what amount of movement would have occurred if surface buoys had been attached to the traps.

Gittings traps that were successfully deployed readily recruited lionfish from nearby artificial reefs (**Fig. 2**). However, lionfish escapement was indicated with 56% of the lionfish that had recruited to traps escaping during retrieval. The proportion of lionfish that escaped did not appear to correlate with higher recruitment of lionfish to traps (see R code for scatter plots). Because traps closed during ascent when SCUBA divers filled lift bags attached to traps with air, trap closing took approximately 3–5 seconds and divers often observed lionfish swimming out in this time. Native species were not observed escaping during trap closing; however, detection of native fishes was an apparent issue.

## Lionfish and native species trap recruitment

A total of 327 lionfish recruited (i.e., number of fish observed within the trap footprint) to Gittings traps during 82 trap sets (n = 58 deployments including paired sets) with 141 lionfish caught (**Fig. 4, left**). Trap bycatch of native species consisted of 28 individuals recruiting to the traps and 29 individuals caught from nine different species (**Fig. 4, right**). This result, where one more native species reef fish was caught (i.e., landed on the boat from the trap pull) versus documented as recruited, indicates an apparent detection error by SCUBA divers. Four native fishes captured in traps were regulated species: two scamp (*Mycteroperca phenax*), one Gulf flounder (*Paralichthys albiguttata)*, and one blue angelfish (*Holacanthus bermudensis*). Additional native species catches consisted of 15 sand perch (*Diplectrum formosum)*, four tomtate (*Haemulon aurolineatum*), two bank sea bass (*Centropristis ocyurus*), two porgies (*Calamus* spp.), one soapfish (*Rypticus* spp.), and one polka-dot batfish (*Ogcocephalus radiatus*). Native species TL measurements were similar to lionfish with a range of 151–350 mm.

**Fig 4. Number and species of fishes in the Gittings traps.** Total counts of lionfish caught, lionfish escaped, and native reef fish species caught in traps deployed near northern Gulf of Mexico artificial reefs during 82 trap deployments. Size of pie slices correspond to the proportion of overall count. Fish images are not drawn to scale.

Lionfish recruitment ranged from 0 to 20 per trap and lionfish catch ranged from 0 to 14 per trap (video of a Gittings trap with high recruitment is provided at <https://youtu.be/1vzByPMm7hQ>). QQ plots indicated that the error structures for the recruitment and catch models (Eq. 1) were best fit with a negative binomial distribution, which is typical for count data [52]. GLMM results indicated lionfish recruitment and catch were significantly affected by distance from the adjacent artificial reef and soak time (**Table 3**). Mean lionfish recruitment to paired traps was 44% lower per trap compared to single traps, but the difference was not significant (P = 0.195) due to the high variance in the data (**Fig. 5A**). Traps placed at close (5 m) and intermediate distances and (15 m) had similar recruitment (P = 0.935), while traps placed far (65 m) from a reef attracted almost no lionfish (P < 0.001) (**Fig. 5B**). Lionfish recruitment was highest for traps deployed for 1 day (**Fig. 5C**). Lionfish catch was 84% lower for the longest soak time of 12–14 days (P = 0.001) while lionfish recruitment for 12–14 days was 39% lower (**Table 3**). Lionfish density on the artificial reef study sites ranged from 6 to 36 fish per 100 m². Unexpectedly, lionfish density on the adjacent artificial reefs was not a significant covariate in predicting lionfish recruitment (P = 0.152) nor lionfish catch (P = 0.267). Mean native species recruitment and catch were <1 fish per trap (**Fig. 5**) and the GLMM results indicated native species recruitment and catch were significantly affected by trap number and soak time (**Table 2**). Paired traps had approximately 75% lower recruitment and catch per trap than single traps (**Fig. 5A, Table 2**). In contrast to the lionfish models where recruitment and catch were lower with longer soak times, recruitment and catch for native fishes increased during soak times of 4–5 day, 8 day and 12–14 day (**Fig. 5C**). Longer soak times predicted 4–12X higher recruitment and catch of native species, although only the 4–5 day level in the native species recruitment model was significant (P = 0.013).

**Table 3. Generalized mixed model results testing deployment factors on lionfish and native species recruitment and catches by Gittings traps.** Effect of deployment factors and lionfish density on mean recruitment (fish observed within the trap footprint during retrieval) and catches (fish landed aboard the vessel) of lionfish and native reef fish species by Gittings traps. Factors examined included number of traps (single or paired), distance from the adjacent artificial reef (5 m, 15 m, or 65 m), soak time (0.25 days, 1 day, 4–5 days, or 12–14 days), and adjacent reef lionfish density (fish per 100 m^2^). Differences were tested with generalized linear mixed models (GLMM) fit with negative binomial error distributions and reef site as a random effect. GLMM outputs show the log-linked parameter estimates for mean fish count recruited or caught per trap, associated 95% confidence intervals (CI) around mean count per trap, and the odds ratio (proportional effect). Odds ratio and hypothesis testing (z- and P-values) represent the difference from the GLMM intercept, i.e., the difference compared to a single trap deployed 5 m from the reef with a soak time of 0.25 days (recruitment model intercept) or 1 day (catch model intercept).

| **Model** | **Factor** | **Level** | **Odds ratio** | **Mean count** | **95% CI** | **z** | **P*** |
| --- | --- | --- | --- | --- | --- | --- | --- |
| Lionfish recruitment | *(Intercept)* |  |  | 2.77 | 1.22-6.33 | 6.33 | **0.015** |
|  | Trap number | Paired | 0.56 | 1.55 | 0.36-2.09 | 2.09 | 0.195 |
|  | Distance | 15 m | 0.95 | 2.63 | 1.47-4.23 | 4.23 | 0.860 |
|  | Distance | 65 m | 0.03 | 0.08 | 0.00-0.02 | 0.02 | **0.001** |
|  | Soak time | 1 day | 1.89 | 5.24 | 5.34-18.2 | 18.2 | **0.042** |
|  | Soak time | 4-5 days | 1.82 | 5.04 | 4.74-17.8 | 17.8 | 0.078 |
|  | Soak time | 8 days | 1.66 | 4.60 | 3.40-17.2 | 17.2 | 0.217 |
|  | Soak time | 12-14 days | 1.50 | 4.16 | 3.16-12.3 | 12.3 | 0.245 |
|  | LF density |  | 1.02 | 2.83 | 2.80-3.00 | 3.00 | 0.152 |
| Lionfish catch | *(Intercept)* |  |  | 4.38 | 1.64-11.7 | 11.7 | **0.003** |
|  | Trap number | Paired | 0.52 | 2.28 | 0.57-2.46 | 2.46 | 0.080 |
|  | Distance | 15 m | 0.76 | 3.33 | 1.30-4.93 | 4.93 | 0.419 |
|  | Distance | 65 m | 0.06 | 0.26 | 0.00-0.13 | 0.13 | **0.009** |
|  | Soak time | 4-5 days | 1.09 | 4.77 | 2.34-11.6 | 11.6 | 0.837 |
|  | LF density | 8 days | 0.31 | 1.36 | 0.11-1.54 | 1.54 | 0.076 |
|  | Soak time | 12-14 days | 0.16 | 0.70 | 0.03-0.34 | 0.34 | **0.001** |
|  | LF density |  | 1.02 | 4.47 | 4.38-4.74 | 4.74 | 0.331 |
| Native species recruitment | *(Intercept)* |  |  | 0.26 | 0.06-1.13 | 1.13 | 0.073 |
|  | Trap number | Paired | 0.23 | 0.06 | 0.00-0.05 | 0.05 | **0.018** |
|  | Distance | 15 m | 2.05 | 0.53 | 0.46-2.62 | 2.62 | 0.107 |
|  | Distance | 65 m | 0.00 | 0.00 | 0.00-NaN | NA | 1.000 |
|  | Soak time | 1 day | 0.36 | 0.09 | 0.00-0.32 | 0.32 | 0.383 |
|  | Soak time | 4-5 days | 4.73 | 1.23 | 1.67-20.2 | 20.2 | **0.014** |
|  | Soak time | 8 days | 2.47 | 0.64 | 0.20-12.4 | 12.4 | 0.389 |
|  | Soak time | 12-14 days | 1.62 | 0.42 | 0.13-3.43 | 3.43 | 0.559 |
|  | LF density |  | 1.00 | 0.26 | 0.25-0.27 | 0.27 | 0.866 |
| Native species catch | *(Intercept)* |  |  | 0.23 | 0.03-1.95 | 1.95 | 0.178 |
|  | Trap number | Paired | 0.26 | 0.06 | 0.00-0.05 | 0.05 | **0.024** |
|  | Distance | 15 m | 0.80 | 0.18 | 0.06-0.37 | 0.37 | 0.642 |
|  | Distance | 65 m | 0.14 | 0.03 | 0.00-0.08 | 0.08 | 0.185 |
|  | Soak time | 4-5 days | 6.39 | 1.47 | 1.22-72.0 | 72.0 | 0.074 |
|  | Soak time | 8 days | 4.98 | 1.15 | 0.53-61.6 | 61.6 | 0.185 |
|  | Soak time | 12-14 days | 6.55 | 1.51 | 1.24-79.1 | 79.1 | 0.076 |
|  | LF density |  | 0.98 | 0.23 | 0.21-0.24 | 0.24 | 0.454 |
| *P-values <0.05 are bolded. | | | | | | | |

**Fig 5. Lionfish and native species recruitment.** Mean (± 95 CI) count of lionfish and native species recruitment (fish observed within the trap footprint during retrieval) per trap with the number of replicates per level indicated (n). Trap deployment configurations examined factors of A) distance to the adjacent artificial reef, B) number of traps, and C) soak time.

Size distributions were multi-modal for both lionfish caught by traps (n = 137) and lionfish speared (n = 3,063) from nGOM artificial reefs (**Fig. 6**), likely due to distinct TL modes for juvenile and adult lionfish [53,54]. Lionfish sizes (measured in TL) were not significantly different between lionfish captured from traps deployed at distances 5 m and 15 m (**Fig. 6A,** KS test, P = 0.591). Lionfish size from 65 m distanced traps were not compared as only one lionfish was caught. Probability density plots showed traps appear to have caught disproportionately fewer juvenile (<200 mm) and more large adult (>300 mm) lionfish compared to catches from spearfishing (**Fig. 6B**) and results from the KS test indicate sizes of trap-captured lionfish were significantly larger than speared lionfish (**Fig. 6B**, P = 0.006). Mean TL for trap-caught lionfish (277 mm) was 19 mm larger than spear-caught lionfish (296 mm). Given a weight-length relationship for nGOM lionfish of *W* = 3.09E-6 x (*TL*)^3.26 [55]**,** the mean weight of trap-caught lionfish were estimated to be 73 grams larger.

**Fig 6. Lionfish size distributions by distance to reef and harvest gear.** A) Lionfish total length (TL) probability density distributions for lionfish captured in Gittings traps for traps deployed at near (5 m) and intermediate (15 m) distances to the adjacent artificial reef. B) Lionfish TL distributions for lionfish harvested by Gittings traps (all treatments) and lionfish sampled concurrently (June–December 2018) by spearfishing on similar northern Gulf of Mexico artificial reefs. Mean TL per distribution is indicated by a vertical dashed line.

# DISCUSSION

The lack of apparent environmental impacts demonstrates traps may be suitable for further field testing. Lionfish recruitment and catch were >10X higher than that of native species and traps did not move during severe weather events, although traps will need further testing for their potential to move when they have surface buoys. Given that wide-scale commercial use of Gittings traps would entail remote deployment and recovery from the surface, design iterations and field tests will be necessary to improve deployment success and lionfish catch rates. Potential gear modifications could include the following: adjustments to flotation and ballast that could help traps keep a vertical orientation during descent to allow successful opening, a reconfigured harness that closes the jaws more quickly and keeps them closed during recovery, a looser net that could allow more billowing and not contact lionfish during closure, and faster trap retrieval provided by a shipboard winch. Trap mass will also be a consideration for commercial application. The traps in this study were built with relatively thick #6 rebar and a trap had a mass of ~35 kg, which made it difficult for a single person to move or deploy them. Moreover, the cost to bend this thick of rebar into the semi-circular jaw frames constituted >50% of their construction costs. Either #4 rebar (13 mm / 0.5 in diameter) or #5 rebar (15.875 mm / 0.625” diameter), which are both more easily sourced and manipulated, may provide better materials for trap jaws. Concurrent work has also examined design and material modifications for reducing production costs of Gittings traps with an octagon design rather than a circular one (pers. comm., S. Delello, ReefSave).

Removal efficiency by Gittings traps on artificial reefs was 12–26% of the lionfish observed on adjacent artificial reefs, which is higher than many fisheries where removal efficiency is <10% [56–58]. However, removal efficiencies and uncaptured lionfish would need to be considered when evaluating the potential community benefits offered by lionfish trapping. Lionfish removals do not necessarily translate into ecological benefits [14,59,60] given that lionfish predation [61,62], growth [55,63], and colonization [14,51,60] rates are controlled via density-dependent feedbacks. Ecosystem models may thus be appropriate for examining the potential community effects of a deepwater lionfish fishery [15,64]. Removal efficiency by traps were also considerably lower than the >85% removal efficiency estimated for spearfishing lionfish on nGOM artificial reefs [46]. Considering that spearfishing can reduce lionfish densities in areas frequented by divers [65–67] and that spearfishing fisheries have caused severe depletion of other reef fishes [68–70], we expect it to remain the most efficient and cost-effective method for removing lionfish biomass at depths <40 m.

Lionfish catch was potentially optimized by deploying a single trap 15 m or less from a reef site for 1 day. Mean recruitment with this deployment configuration was approximately 5 lionfish and 0.1 native reef fish per trap. Higher lionfish recruitment to traps deployed closer to reefs was expected given lionfish exhibit high site fidelity [71] and central-place foraging [13]. More sampling at distances between 15 and 65 m could be conducted to better understand the effective attraction distance of traps and the linear or non-linear characteristics of this relationship. Higher lionfish recruitment and catches from single versus paired trap deployments also disproved our hypothesis that paired deployments may synergistically recruit more lionfish, considering lionfish are often found in groups [72] and aggregating behavior is driven by broad-scale habitat complexity [73,74]. Tests of alternative soak times indicated that a 1-day deployment had the highest recruitment of lionfish and lowest recruitment of native species, although results were not significantly higher due to the high variation in the results. Despite this variation, it appears to indicate that longer soak times may be unnecessary. This may also suggest that lionfish recruit relatively quickly to traps but leave during longer soak times, while native fish may take longer to recruit to traps. Our observations of higher lionfish and lower native species recruitment during shorter soak times, and the converse for longer soak times, supports the idea that lionfish presence deters or excludes native reef fishes, which has been reported in other studies on the indirect effects of lionfish on native species [75–78].

Overall lionfish recruitment numbers to the Gittings trap were about double the numbers observed on lobster traps deployed near mesophotic reefs in Bermuda [34]. Subsequent experimental tests on Bermudan reefs with modified lobster trap funnels found that, similar to this study, lionfish catches were also highly variable and right-skewed with mean recruitment to the lobster trap structure ~3 lionfish per trap and ranging to >15 lionfish per trap [79]. While this variation was likely driven by the spatial variation observed in lionfish distributions on Bermuda reefs [9,80], our results unexpectedly indicated that lionfish density on an adjacent artificial reef was not a significant predictor of lionfish recruitment or catch by traps. We had anticipated that lionfish movement from reefs to traps would correlate to site density given that high lionfish densities have been related to local prey depletion [12,14,81], cannibalism [82,83], lower body condition [55], and greater movement on coral reefs [61,84].

A possible selectivity demonstrated by Gittings traps for larger lionfish could have implications for depleting their populations. Smaller lionfish may have escaped from the traps at greater rates and, in retrospect, it would have been useful to collect size estimates of the lionfish by divers prior to trap retrieval. Alternatively, traps may attract larger individuals. Higher movement rates for larger lionfish is reasonable as they are more physically capable to make such movements and face lower risk of predation in transit [85,86]. Juvenile lionfish risk conspecific predation [82,87], particularly in areas of high lionfish density [83] where this study was conducted, and smaller individuals may thus occupy smaller foraging arenas near reefs [88]. Given fecundity increases exponentially with length [89–91], a gear selectivity bias toward larger fish may have a greater impact on reducing lionfish egg production. High enough reductions of large spawners would lead to recruitment overfishing whereby the reduced spawning stock decreases future population growth [92]. That said, removal efforts aimed to deplete lionfish biomass should also target juveniles. Harvest of faster-growing juveniles [54,55,93] contributes to growth overfishing [94], and age-structured lionfish population models indicate controlling lionfish population growth requires removing smaller individuals [23,24].

The potential for lionfish traps or other novel harvest technologies to reduce lionfish biomass will ultimately be contingent on their capacity to harvest them from natural reefs. Natural reefs constitute over 99% of the region’s reef habitat, with 90% being mesophotic reefs deeper than 40 m [95]. Even on shallower natural reefs accessible to divers, spearfishing catch rates are limited by lower lionfish density [3,55] and lower removal efficiency [46]. Similar gear testing to that conducted in this study will be needed for traps deployed near natural reefs to evaluate their potential for habitat damage or bycatch considering the differences in benthic structure and community composition [14]. It is also unknown whether lionfish attraction will be higher for traps near natural versus artificial reefs. Lionfish densities on nGOM artificial reefs are >10X higher than on nGOM natural reefs [3,46,55], which suggests a trap structure may readily attract lionfish from natural reefs. However, if lionfish movement and home range are largely driven by density-dependence or food availability [14,61,84], then the substantially lower densities on natural reefs may critically reduce catch rates. The potential economic viability of a lionfish trap fishery should be carefully considered. Future trap field testing could examine design strategies to increase lionfish attraction (e.g., using lights, sound, or different structures) and technoeconomic assessments may identify capital and operational expenses to evaluate what catch rates could make lionfish trapping commercially feasible.

# Acknowledgements

We thank Josh and Joe Livingston (Dreadknot Charters), Kara Wall (Florida Fish and Wildlife Research Institute), Tony Reyer (NOAA), Sal DeLello (Reef Save), Laura Tiu (Florida Sea Grant), Stacy Frank (Lionfish University), Jim Hart (Lionfish University), Alexandria Tucker (University of Florida), Dominic Andradi-Brown (Universiy of Oxford), and Florida Fish and Wildlife Conservation Commission personnel Alan Pierce, Kali Spurgin, Amy Brower, Hanna Tillotson, and Michael Kennison.

# References

1.	Schofield PJ. Update on geographic spread of invasive lionfishes (*Pterois volitans* [Linnaeus, 1758] and *P. miles* [Bennett, 1828]) in the Western North Atlantic Ocean, Caribbean Sea and Gulf of Mexico. Aquat Invasions. 2010;5: 117–122. doi:10.3391/ai.2010.5.S1.024

2.	Azzurro E, Stancanelli B, Di Martino V, Bariche M. Range expansion of the common lionfish *Pterois miles* (Bennett, 1828) in the Mediterranean Sea: an unwanted new guest for Italian waters. BioInvasions Rec. 2017;6. doi:10.3391/bir.2017.6.2.01

3.	Dahl KA, Patterson WF. Habitat-specific density and diet of rapidly expanding invasive red lionfish, *Pterois volitans*, populations in the northern Gulf of Mexico. PLoS One. 2014;9: e105852. doi:10.1371/journal.pone.0105852

4.	Claydon JAB, Calosso MC, Traiger SB. Progression of invasive lionfish in seagrass, mangrove and reef habitats. Mar Ecol Prog Ser. 2012;448: 119–129. doi:10.3354/meps09534

5.	Barbour AB, Montgomery ML, Adamson AA., Díaz-Ferguson E, Silliman BR. Mangrove use by the invasive lionfish *Pterois volitans*. Mar Ecol Prog Ser. 2010;401: 291–294. doi:10.3354/meps08373

6.	Jud Z, Layman C, Lee J, Arrington D. Recent invasion of a Florida (USA) estuarine system by lionfish *Pterois volitans / P. miles*. Aquat Biol. 2011;13: 21–26. doi:10.3354/ab00351

7.	Lesser MP, Slattery M. Phase shift to algal dominated communities at mesophotic depths associated with lionfish (*Pterois volitans*) invasion on a Bahamian coral reef. Biol Invasions. 2011;13: 1855–1868. doi:10.1007/s10530-011-0005-z

8.	Andradi-Brown DA, Vermeij MJA, Slattery M, Lesser M, Bejarano I, Appeldoorn R, et al. Large-scale invasion of western Atlantic mesophotic reefs by lionfish potentially undermines culling-based management. Biol Invasions. 2017;19: 939–954. doi:10.1007/s10530-016-1358-0

9.	Goodbody-Gringley G, Eddy C, Pitt JM, Chequer AD, Smith SR. Ecological drivers of invasive lionfish (*Pterois volitans* and *Pterois miles*) distribution across mesophotic reefs in Bermuda. Front Mar Sci. 2019;6: 258. doi:10.3389/fmars.2019.00258

10.	Gress E, Andradi-Brown DA, Woodall L, Schofield PJ, Stanley K, Rogers AD. Lionfish (*Pterois* spp.) invade the upper-bathyal zone in the western Atlantic. PeerJ. 2017;5: e3683. doi:10.7717/peerj.3683

11.	Green SJ, Côté IM. Record densities of Indo-Pacific lionfish on Bahamian coral reefs. Coral Reefs. 2009;28: 107–107. doi:10.1007/s00338-008-0446-8

12.	Green SJ, Akins JL, Maljković A, Côté IM. Invasive lionfish drive Atlantic coral reef fish declines. Goldstien SJ, editor. PLoS One. 2012;7: e32596. doi:10.1371/journal.pone.0032596

13.	Benkwitt CE. Central-place foraging and ecological effects of an invasive predator across multiple habitats. Ecology. 2016;97: 2729–2739. doi:10.1002/ecy.1477

14.	Dahl KA, Patterson III WF, Snyder RA. Experimental assessment of lionfish removals to mitigate reef fish community shifts on northern Gulf of Mexico artificial reefs. Mar Ecol Prog Ser. 2016;558: 207–221. doi:10.3354/meps11898

15.	Chagaris D, Binion-Rock S, Bogdanoff A, Dahl K, Granneman J, Harris HE, et al. An ecosystem-based approach to evaluating impacts and management of invasive lionfish. Fisheries. 2017;42: 421–431. doi:10.1080/03632415.2017.1340273

16.	Albins MA, Hixon MA. Worst case scenario: potential long-term effects of invasive predatory lionfish (*Pterois volitans*) on Atlantic and Caribbean coral-reef communities. Environ Biol Fishes. 2013;96: 1151–1157. doi:10.1007/s10641-011-9795-1

17.	Hixon MA, Green SJ, Albins MA, Akins JL, Morris JA. Lionfish: a major marine invasion. Mar Ecol Prog Ser. 2016;558: 161–165. doi:10.3354/meps11909

18.	Valdivia A, Bruno JF, Cox CE, Hackerott S, Green SJ. Re-examining the relationship between invasive lionfish and native grouper in the Caribbean. PeerJ. 2014;2: e348. doi:10.7717/peerj.348

19.	Hackerott S, Valdivia A, Green SJ, Côté IM, Cox CE, Akins L, et al. Native predators do not influence invasion success of Pacific lionfish on Caribbean reefs. Guichard F, editor. PLoS One. 2013;8: e68259. doi:10.1371/journal.pone.0068259

20.	Anton A, Simpson MS, Vu I. Environmental and biotic correlates to lionfish invasion success in Bahamian coral reefs. PLoS One. 2014;9: e106229. doi:10.1371/journal.pone.0106229

21.	Morris JA, Akins JL, Green SJ, Buddo DSA, Lozano RG. Invasive Lionfish: A Guide to Control and Management. Morris JA, editor. Gulf and Caribbean Fisheries Institute Special Publication Series. Marathon, FL: Gulf and Caribbean Fisheries Institute, Inc.; 2012.

22.	Sutherland WJ, Barnard P, Broad S, Clout M, Connor B, Côté IM, et al. A 2017 horizon scan of emerging issues for global conservation and biological diversity. Trends in Ecology and Evolution. Elsevier Ltd; 2017. pp. 31–40. doi:10.1016/j.tree.2016.11.005

23.	Barbour AB, Allen MS, Frazer TK, Sherman KD. Evaluating the potential efficacy of invasive lionfish (*Pterois volitans*) removals. PLoS One. 2011;6. doi:10.1371/journal.pone.0019666

24.	Morris JA, Shertzer KW, Rice JA. A stage-based matrix population model of invasive lionfish with implications for control. Biol Invasions. 2011;13: 7–12. doi:10.1007/s10530-010-9786-8

25.	Johnston M, Purkis SJ. A coordinated and sustained international strategy is required to turn the tide on the Atlantic lionfish invasion. Mar Ecol Prog Ser. 2015;533: 219–235. doi:10.3354/meps11399

26.	Johnston MA, Gittings SR, Morris JA. NOAA national marine sanctuaries lionfish response plan (2015-2018): responding, controlling, and adapting to an active marine invasion. 2015. Available: https://repository.library.noaa.gov/view/noaa/13954

27.	GEBCO. GEBCO 2019 Grid. 2019. doi:doi:10.5285/836f016a-33be-6ddc-e053-6c86abc0788e

28.	Andradi-Brown DA. Invasive Lionfish (*Pterois volitans* and *P. miles*): Distribution, Impact, and Management. Mesophotic Coral Ecosystems. Springer; 2019. pp. 931–941. doi:10.1007/978-3-319-92735-0_48

29.	Nuttall M, Johnston MA, Eckert RJ, Embesi JA, Hickerson EL, Schmahl GP. Lionfish (*Pterois volitans* [Linnaeus, 1758] and *P. miles* [Bennett, 1828]) records within mesophotic depth ranges on natural banks in the Northwestern Gulf of Mexico. BioInvasions Rec. 2014;3: 111–115. doi:10.3391/bir.2014.3.2.09

30.	Switzer TS, Tremain DM, Keenan SF, Stafford CJ, Parks SL, McMichael RH. Temporal and spatial dynamics of the lionfish invasion in the eastern Gulf of Mexico: perspectives from a broadscale trawl survey. Mar Coast Fish. 2015;7: 1–8. doi:10.1080/19425120.2014.987888

31.	Reed J, Farrington S, Harter S, Moe H, Hanisak D, David A. Characterization of the mesophotic benthic habitat and fish assemblages from ROV dives on Pulley Ridge and Tortugas during 2014 R/V Walton Smith cruise. 2015. Available: https://repository.library.noaa.gov/view/noaa/17810

32.	Andradi-Brown DA, Grey R, Hendrix A, Hitchner D, Hunt CL, Gress E, et al. Depth-dependent effects of culling—do mesophotic lionfish populations undermine current management? R Soc Open Sci. 2017;4: 170027. doi:10.1098/rsos.170027

33.	Johnston MW, Purkis SJ. Hurricanes accelerated the Florida-Bahamas lionfish invasion. Glob Chang Biol. 2015;21: 2249–2260. doi:10.1111/gcb.12874

34.	Pitt JM, Trott TM. Efforts to develop a lionfish-specific trap for use in Bermuda waters. Proceedings of the 66th Gulf and Caribbean Fisheries Institute. Corpus Cristi, TX; 2013. pp. 187–190.

35.	Wang Y. Remote operated vehicle for selectively harvesting target species. United States; US10392085B2, 2018.

36.	Gittings SR. Apparatus for harvesting lionfish. United States; US20190021297A1, 2018. Available: https://patents.google.com/patent/US20190021297A1/en

37.	Gittings SR. Encouraging results in tests of new lionfish trap design. 69th Gulf and Caribbean Fisheries Institute. 2016. pp. 206–214.

38.	Gittings SR, Fogg AQ, Frank S, Hart J V, Clark A, Clark B, et al. Going deep for lionfish: designs for two new traps for capturing lionfish in deep water. Silver Spring, MD; 2017. doi:10.13140/RG.2.2.22898.50886

39.	Bogdanoff AK, Akins JL, Morris JA, Workgroup 2013 GCFI Lionfish. Invasive lionfish in the marketplace: challenges and opportunities. Proceedings of the 66th Gulf and Caribbean Fisheries Institute. 2014. pp. 140–147.

40.	Blakeway RD, Jones GA, Boekhoudt B. Controlling lionfishes (*Pterois* spp.) with consumption: survey data from Aruba demonstrate acceptance of non-native lionfishes on the menu and in seafood markets. Fish Manag Ecol. 2019; fme.12404. doi:10.1111/fme.12404

41.	Chapman JK, Anderson LG, Gough CLA, Harris AR. Working up an appetite for lionfish: a market-based approach to manage the invasion of *Pterois volitans* in Belize. Mar Policy. 2016;73: 256–262. doi:10.1016/J.MARPOL.2016.07.023

42.	McClanahan TR, Mangi SC. Gear-based management of a tropical artisanal fishery based on species selectivity and capture size. Fish Manag Ecol. 2004;11: 51–60. doi:10.1111/j.1365-2400.2004.00358.x

43.	Loh TL, McMurray SE, Henkel TP, Vicente J, Pawlik JR. Indirect effects of overfishing on Caribbean reefs: sponges overgrow reef-building corals. PeerJ. 2015;2015: e901. doi:10.7717/peerj.901

44.	Lamberte A, Swingle WE. Amendments to the reef fish fishery management plan for the reef fish resources of the Gulf of Mexico. 1993. doi:10.25923/XGHH-CR28

45.	Collier C, Cheuvront B, Blough H, Waugh G, McGovern J, MacLauchlin K, et al. Regulatory amendment 16: changes to the seasonal slosure for the black sea bass pot sector. 2016. doi:10.25923/2WEY-PQ14

46.	Harris HE, Patterson III WF, Ahrens RNM, Allen MS. Detection and removal efficiency of invasive lionfish in the northern Gulf of Mexico. Fish Res. 2019;213: 22–32. doi:10.1016/j.fishres.2019.01.002

47.	Patterson III WF, Dance MA, Addis DT. Development of a remotely operated vehicle based methodology to estimate fish community structure at artificial reef sites in the northern Gulf of Mexico. Proceedings of the 61st Gulf and Caribbean Fisheries Institute. 2009. pp. 263–270.

48.	Bolker BM, Brooks ME, Clark CJ, Geange SW, Poulsen JR, Stevens MHH, et al. Generalized linear mixed models: a practical guide for ecology and evolution. Trends Ecol Evol. 2009;24: 127–135. doi:10.1016/J.TREE.2008.10.008

49.	Bates D, Mächler M, Bolker B, Walker S. Fitting linear mixed-effects models using lme4. J Stat Softw. 2015. doi:10.18637/jss.v067.i01

50.	Venables WN, Ripley BD. Modern Applied Statistics with S. 4th ed. New York: Springer; 2002. Available: http://www.stats.ox.ac.uk/pub/MASS4

51.	Harris HE, Fogg AQ, Allen MS, Ahrens RNM, Patterson WF. Precipitous declines in northern Gulf of Mexico invasive lionfish populations following the emergence of an ulcerative skin disease. Sci Rep. 2020;10: 1–17. doi:10.1038/s41598-020-58886-8

52.	Bolker BM. Ecological Models and Data in R. Princeton University Press; 2007. doi:10.1086/644667

53.	Johnson EG, Swenarton MK. Age, growth and population structure of invasive lionfish (*Pterois volitans/miles*) in northeast Florida using a length-based, age-structured population model. PeerJ. 2016;4: e2730. doi:10.7717/peerj.2730

54.	Fogg AQ, Ingram W, Peterson MS, Brown-Peterson NJ. Comparing age and growth patterns of invasive lionfish among three ecoregions of the northern Gulf of Mexico. 2015 [cited 12 Mar 2018]. doi:10.13140/RG.2.1.1425.3525

55.	Dahl K, Edwards M, Patterson III WF. Density-dependent condition and growth of invasive lionfish in the northern Gulf of Mexico. Mar Ecol Prog Ser. 2019;623: 145–159. doi:10.3354/meps13028

56.	Askey PJ, Richards SA, Post JR, Parkinson EA. Linking angling catch rates and fish learning under catch-and-release regulations. North Am J Fish Manag. 2006;26: 1020–1029. doi:10.1577/M06-035.1

57.	Salthaug A, Aanes S. Catchability and the spatial distribution of fishing vessels. Can J Fish Aquat Sci. 2003;60: 259–268. doi:10.1139/f03-018

58.	Hangsleben MA, Allen MS, Gwinn DC. Evaluation of electrofishing catch per unit effort for indexing fish abundance in Florida lakes. Trans Am Fish Soc. 2013;142: 247–256. doi:10.1080/00028487.2012.730106

59.	Harms-Tuohy C, Appeldoorn R, Craig M. The effectiveness of small-scale lionfish removals as a management strategy: effort, impacts and the response of native prey and piscivores. Manag Biol Invasions. 2018;9: 149–162. doi:10.3391/mbi.2018.9.2.08

60.	Smith NS, Green SJ, Akins JL, Miller S, Côté IM. Density-dependent colonization and natural disturbance limit the effectiveness of invasive lionfish culling efforts. Biol Invasions. 2017;19: 2385–2399. doi:10.1007/s10530-017-1449-6

61.	Benkwitt C. Invasive lionfish increase activity and foraging movements at greater local densities. Mar Ecol Prog Ser. 2016;558: 255–266. doi:10.3354/meps11760

62.	Benkwitt CE. Non-linear effects of invasive lionfish density on native coral-reef fish communities. Biol Invasions. 2015;17: 1383–1395. doi:10.1007/s10530-014-0801-3

63.	Benkwitt CE. Density-dependent growth in invasive lionfish (*Pterois volitans*). Steinke D, editor. PLoS One. 2013;8: e66995. doi:10.1371/journal.pone.0066995

64.	Arias-González JE, González-Gándara C, Luis Cabrera J, Christensen V. Predicted impact of the invasive lionfish *Pterois volitans* on the food web of a Caribbean coral reef. Environ Res. 2011;111: 917–925. doi:10.1016/j.envres.2011.07.008

65.	De León R, Vane K, Bertuol P, Chamberland VC. Effectiveness of lionfish removal efforts in the southern Caribbean. Endanger Species Res. 2013;22: 175–182. doi:10.3354/esr00542

66.	Frazer TK, Jacoby CA, Edwards MA, Barry SC, Manfrino CM. Coping with the lionfish invasion: can targeted removals yield beneficial effects? Rev Fish Sci. 2012;20: 185–191. doi:10.1080/10641262.2012.700655

67.	Usseglio P, Selwyn JD, Downey-Wall AM, Hogan JD. Effectiveness of removals of the invasive lionfish: how many dives are needed to deplete a reef? PeerJ. 2017;5: e3043. doi:10.7717/peerj.3043

68.	Giglio VJ, Bender MG, Zapelini C, Ferreira CEL. The end of the line? Rapid depletion of a large-sized grouper through spearfishing in a subtropical marginal reef. Perspect Ecol Conserv. 2017;15: 115–118. doi:10.1016/j.pecon.2017.03.006

69.	Frisch AJ, Cole AJ, Hobbs J-PA, Rizzari JR, Munkres KP. Effects of spearfishing on reef fish populations in a multi-use conservation area. Ferse SCA, editor. PLoS One. 2012;7: e51938. doi:10.1371/journal.pone.0051938

70.	Godoy N, Gelcich S, Vasquez J a., Castilla JC. Spearfishing to depletion: evidence from temperate reef fishes in Chile. Ecol Appl. 2010;20: 1504–1511. doi:10.1890/09-1806.1

71.	Jud ZR, Layman CA. Site fidelity and movement patterns of invasive lionfish, *Pterois* spp., in a Florida estuary. J Exp Mar Bio Ecol. 2012;414–415: 69–74. doi:10.1016/j.jembe.2012.01.015

72.	García-Rivas MDC, Machkour-M’Rabet S, Pérez-Lachaud G, Schmitter-Soto JJ, Doneys C, St-Jean N, et al. What are the characteristics of lionfish and other fishes that influence their association in diurnal refuges? Mar Biol Res. 2017;13: 899–908. doi:10.1080/17451000.2017.1314496

73.	Hunt CL, Kelly GR, Windmill H, Curtis-Quick J, Conlon H, Bodmer MD V., et al. Aggregating behaviour in invasive Caribbean lionfish is driven by habitat complexity. Sci Rep. 2019;9. doi:10.1038/s41598-018-37459-w

74.	Davis A. Integrating remote sensing and diver observations to predict the distribution of invasive lionfish on Bahamian coral reefs. Mar Ecol Prog Ser. 2019;623: 1–11. doi:10.3354/meps13067

75.	Lewis JP, Tarnecki JH, Garner SB, Chagaris DD, Patterson WF. Changes in reef fish community structure following the Deepwater Horizon oil spill. Sci Rep. 2020;10: 5621. doi:10.1038/s41598-020-62574-y

76.	Tarnecki JH, Patterson WF. Changes in Red Snapper Diet and Trophic Ecology Following the Deepwater Horizon Oil Spill. Mar Coast Fish. 2015;7: 135–147. doi:10.1080/19425120.2015.1020402

77.	Marshak AR, Heck KL, Jud ZR. Ecological interactions between Gulf of Mexico snappers (*Teleostei: Lutjanidae*) and invasive red lionfish (*Pterois volitans*). PLoS One. 2018;13: e0206749. doi:10.1371/journal.pone.0206749

78.	Ellis R, Faletti M. Native grouper indirectly ameliorates the negative effects of invasive lionfish. Mar Ecol Prog Ser. 2016;558: 267–279. doi:10.3354/meps11808

79.	Pitt JM, Trott TM. A lionfish trap for use in Bermuda, with potential applications elsewhere Lionfish in Bermuda. 2015. pp. 2–3. doi:10.13140/RG.2.2.22051.55844

80.	Goodbody-Gringley G, Pitt JM, Eddy WC, Chequer AD, Noyes T, Blanco-Bercial L, et al. Assessment and management of invasive lionfish populations in Bermuda. Proceedings of the 71st Gulf and Caribbean Fisheries Institute. San Andrés, Colombia; 2019. Available: https://www.researchgate.net/publication/337290518_Assessment_and_Management_of_Invasive_Lionfish_Populations_in_Bermuda

81.	Ingeman KE. Lionfish cause increased mortality rates and drive local extirpation of native prey. Mar Ecol Prog Ser. 2016;558. doi:10.3354/meps11821

82.	Dahl KA, Patterson III WF, Robertson A, Ortmann AC. DNA barcoding significantly improves resolution of invasive lionfish diet in the northern Gulf of Mexico. Biol Invasions. 2017;19: 1917–1933. doi:10.1007/s10530-017-1407-3

83.	Dahl KA, Portnoy DS, Hogan JD, Johnson JE, Gold JR, Patterson WF. Genotyping confirms significant cannibalism in northern Gulf of Mexico invasive red lionfish, *Pterois volitans*. Biol Invasions. 2018;20: 3513–3526. doi:10.1007/s10530-018-1791-3

84.	Tamburello N, Côté IM. Movement ecology of Indo-Pacific lionfish on Caribbean coral reefs and its implications for invasion dynamics. Biol Invasions. 2015;17: 1639–1653. doi:10.1007/s10530-014-0822-y

85.	Jenkins DG, Brescacin CR, Duxbury C V., Elliott JA, Evans JA, Grablow KR, et al. Does size matter for dispersal distance? Glob Ecol Biogeogr. 2007;16: 415–425. doi:10.1111/j.1466-8238.2007.00312.x

86.	Lima SL, Dill LM. Behavioral decisions made under the risk of predation: a review and prospectus. Can J Zool. 1990;68: 619–640. doi:10.1139/z90-092

87.	Côté IM, Green SJ, Morris JA, Akins JL, Steinke D. Diet richness of invasive Indo-Pacific lionfish revealed by DNA barcoding. Mar Ecol Prog Ser. 2013;472: 249–256. doi:10.3354/meps09992

88.	Ahrens RNM, Walters CJ, Christensen V. Foraging arena theory. Fish Fish. 2012;13: 41–59. doi:10.1111/j.1467-2979.2011.00432.x

89.	Fogg AQ, Brown-Peterson NJ, Peterson MS. Reproductive life history characteristics of invasive red lionfish (*Pterois volitans*) in the northern Gulf of Mexico. Bull Mar Sci. 2017;93: 791–813. doi:10.5343/bms.2016.1095

90.	Gardner PG, Frazer TK, Jacoby CA, Yanong RPE. Reproductive biology of invasive lionfish (*Pterois* spp.). Front Mar Sci. 2015;2: 7. doi:10.3389/fmars.2015.00007

91.	Denney NH, Jennings S, Reynolds JD. Life–history correlates of maximum population growth rates in marine fishes. Proc R Soc London Ser B Biol Sci. 2002;269: 2229–2237. doi:10.1098/rspb.2002.2138

92.	Hilborn R, Walters CJ. Quantitative Fisheries Stock Assessment: Choice, Dynamics and Uncertainty. Springer US; 1992. doi:10.1007/978-1-4615-3598-0

93.	Fogg AQ, Evans JT, Peterson MS, Brown-Peterson NJ, Hoffmayer ER, Ingram Jr. GW. Comparison of age and growth parameters of invasive red lionfish (*Pterois volitans*) across the northern Gulf of Mexico. Fish Bull. 2019;117: 1–15. doi:10.7755/FB.117.3.1

94.	Allen MS, Ahrens RNM, Hansen MJ, Arlinghaus R. Dynamic angling effort influences the value of minimum-length limits to prevent recruitment overfishing. Fish Manag Ecol. 2013;20: 247–257. doi:10.1111/j.1365-2400.2012.00871.x

95.	Buczkowski BJ, Reid JA, Jenkins CJ, Reid JM, Williams SJ, Flocks JG. usSEABED: Gulf of Mexico and Caribbean (Puerto Rico and U.S. Virgin Islands) offshore surficial sediment data release. Data Ser. 2006. doi:10.3133/DS146

# Additional Information

## Funding

Financial support for this research was provided by the Florida Fish and Wildlife Conservation Commission (Grant No. 13416 to R. N. M. Ahrens and H. E. Harris). Support for H. E. Harris was provided by the National Science Foundation Graduate Research Fellowship Program (Grant Nos. DGE-1315138 and DGE-182473). The funders had no role in study design, data collection and analysis, decision to publish, or preparation of the manuscript. Opinions, findings, or conclusions expressed in this document do not necessarily reflect the views of our supporting organizations.

## Author contributions

HH, AF, and SG conceived this study. Sampling design was developed by HH, AF, SG, and RA. HH and AF conducted field operations and data sampling. HH conducted analyses with guidance from RA, MA, and WP. Tables and figures were produced by HH with help from co-authors. HH wrote the manuscript first draft and oversaw revisions with input from co-authors.

## Data availability

All analyses are reproducible with the data and R code provided in the supplementary material.

## Ethics declarations

The authors declare no competing interests. Spearfishers were informed and consented to information about their catch and effort being used for research purposes. Lionfish collection by researchers followed humane sampling protocol with euthanasia via pithing the brain case, as reviewed and approved by the University of Florida’s Institutional Animal Care and Use Committee (UF IACUC Protocol #201810225).
