# Supplementary material for: Testing the efficacy of lionfish traps in the northern Gulf of Mexico
Source: PLoS One. 2020 Aug 26;15(8):e0230985. doi: 10.1371/journal.pone.0230985 (PMC7449463; doi:10.1371/journal.pone.0230985)
Supplement: Attachment — Submitted filename: Response to reviewers (2nd revision).docx [file pone.0230985.s009.docx]

Second Round Revisions

Article

PONE-D-20-07219

Testing the efficacy of lionfish traps in the northern Gulf of Mexico

Authors

Holden E. Harris, Alexander Q. Fogg, Stephen R. Gittings, Robert N. M. Ahrens, Micheal S. Allen, and William F. Patterson III^2^

Author Response to the Editor

This revision addressed several minor changes requested by Reviewer 2. All requested changes were made, and they improved the manuscript. The most substantial change was the addition of a test to examine whether season could have affected lionfish size distributions, as described below.

We also took this opportunity to make minor changes to language and syntax to help clarify the material. Although we do not consider any changes substantive, we’d like to point out three in particular to the editor:

1. The term “native species” was changed throughout the manuscript and Fig. 5 to “native fish”. This clarifies the term to represent the number of individual fish, rather than the number of different species: e.g., a mean catch of *x* lionfish and *y* native fish (rather than “native species”) per trap.
2. In Table 3, the column heading “Mean count” was changed to “Parameter estimate (#fish / trap)”. This clarifies that the number given is the mean estimated from the GLMM, rather than the observed mean.
3. One citation was also added [96] for a paper that was recently published (lines 392-393): “. . . if lionfish movement and home range are largely driven by density-dependence or food availability [14,61,84,96] . . .”

Review Comments to the Author

Please use the space provided to explain your answers to the questions above. You may also include additional comments for the author, including concerns about dual publication, research ethics, or publication ethics. (Please upload your review as an attachment if it exceeds 20,000 characters)

Reviewer #1: Thank you for comprehensively addressing my previous review comments and updating the manuscript. I have no further comments.

Reviewer #2: The author has done a thorough job of addressing the comments and suggestions by both reviewers and improving the clarity of the methodology and results and expanding the discussion of the manuscript. It is an interesting, focused and well-written manuscript and with very minor revisions it will be acceptable for publication.

Minor comments

Line 105 remove space before “,”

Done.

Line 219 – This sentence as stated appears unfinished as if you are not going to provide more details since it is the end of the section. May add “as described below.” to the end so the reader knows there is more coming in the next section.

Text updated (lines 222-225): “Because traps were closed for ascent by lift bags that divers attached to traps and filled with air, closing took 3–5 seconds and divers often observed lionfish swimming out in this time. Native fish were not observed escaping during trap closing, although detection of native fishes was an apparent issue, as described below.”

Native Fish Recruitment:
Have you considered that the presence of a SCUBA divers in the water prior to retrieval may have influenced the amount on native fish that were observed “recruitment”.
Certain native species would be more likely to swim away as divers approach compared to lionfish (which are relatively unafraid) and therefore never observed by the divers. These individuals may therefore be at risk of being captured by the trap as bycatch if retrieved by the boat without divers. It could be argued that fish skittish of divers would also be skittish of a moving trap and would have likely escaped as it closed. This may be worth discussing briefly.

We think too much discussion of this would be overly speculative. As the reviewer notes, the same avoidance behavior that may cause a fish to leave the trap with divers nearby could also cause them to leave before the trap closes.

We agree it is worth noting the potential for diver effects on the native species catches in this study. The text was updated as follows (lines 386-388): “Remote recovery of traps may also influence bycatch rates and species composition as some native fish could have reacted to divers and left the trap footprint before it closed.”

285-290
The authors have addressed the size issue and edited the results and discussion thoroughly so no addition changes are required.
However, it would be interesting to look at the size distribution of lionfish from the spearfish each month or each season to see if there was larger number of recruiting juveniles captured in June and July by spear and therefore skewing this comparison? Realizing the sample size from the traps is likely not large enough to run this comparison per month but maybe seasonally? June-August and September-December?

Indeed, the potential for seasonal recruitment to skew the results is worth examining and we appreciate the suggested method to do so. In this revision, we tested whether or not mean TL differed between seasons with a nonparametric two-sample Kolmogorov–Smirnov (KS) test, (the same test used for the other differences in size distribution). Seasons were defined as Jun-Sep vs. Nov-Dec given that the data was more balanced and these better represented changes in water temperature, i.e., warmer in late summer (Jun-Sep) and cooler in late-fall-to-early-winter (Nov-Dec). Note that data collection was not performed during October due to impacts from Hurricane Michael.

The KS test indicated TL distributions were not significantly different by month or season, as described in the updated manuscript results. Plots for these are shown here and provided in the revised R-code, but were not added to the manuscript. Brief explanations for the KS test and the results were added to the manuscript methods and results:

Updated methods (lines 190-192): “Given that lionfish recruitment on subtropical reefs varies by season [51,53], TL distributions from trap-caught lionfish was also compared between seasons with a KS test. Seasons were defined as late summer (Jun–Sep) and early winter (Nov–Dec).”

Updated results (lines 295-296): “There was no significant difference in the size distributions for trap-caught lionfish between seasons (Jun–Sep vs. Nov–Dec; KS test, D = 0.192, P = 0.201).”

For easy reference, here are the boxplots for TL by month and by season, which are also provided in the updated R code:

Line 344
As you stated in the sentence above soak time may be the factor in the density of native fish not lionfish presence. Careful not to overstate the effect of lionfish presence on native species density. Maybe it was the opposite and the later recruitment of native species chased the lionfish away?

Four recent studies have shown indirect effects of lionfish presence on native species. Our purpose was to note that our observations were consistent with those findings. We restated the sentence to clarify that our observations were correlative. The language was also hedged to walk-back the conclusiveness of the statement (lines 347-353): “Though these were not significant different from other soak times, it suggests that longer soak times may be unnecessary. It may also suggest that lionfish recruit relatively quickly to traps but gradually leave, while native fish take longer to recruit to traps but tend to stay. The observed inverse correlation between lionfish and native fish abundance, particularly during the early stages of recruitment to the traps, appears consistent with findings from other studies that report indirect effects of lionfish and that their presence deters native fish from occupying reef space [75–78].”

First Round Revisions

Editor Review
Thank you for submitting your manuscript to PLOS ONE. After careful consideration, we feel that it has merit but does not fully meet PLOS ONE’s publication criteria as it currently stands. Therefore, we invite you to submit a revised version of the manuscript that addresses the points raised during the review process.

More specifically, along with the (many) minor and editorial comments the authors should address the following issues. In discussion the significance of the findings of the manuscript should not be overstated. The size effect should be discussed along with the behavior and some details on the size of native fish. Some details on the density of the lionfish populations should be provided.

The manuscript was revised considerably based on the edits and comments from the referees and editor. Addressing these comments and concerns improved the scientific rigor, clarity, and overall presentation of the study. Specifically, our revision provides details and clarification based on the reviewer guidance for the following: describing the study sites, defining lionfish population densities per site, showing the replicates in the statistical analysis, describing observations of fish behavior, discussing detection error for native reef fish species, clarifying the timing for spearfishing sampling, and discussing other lionfish trap research. Furthermore, we better acknowledge our data limitations, the high variance in some of the results, and walk-back the strength of some of the conclusions made in the discussion (e.g., the difference in fish size). Our purpose in this study was to show our work testing an innovative harvest gear for invasive lionfish, consider the potential limitations and applications based on our findings, and discuss directions of continued research and development. Addressing the reviewer comments helped to better achieve these objectives and we believe publishing this revised article in *PLOS ONE* will make a valuable scientific contribution.

Below are our point-by-point responses to the questions, issues, and requested changes by the reviewers. Our response are denoted with blue text. We were able to make nearly all of the requested changes or clarifications from the referees. Any questions or changes that could not be answered are explained or defended. We think the editor will find our revised article suited (or nearly suitable) for publication, and we are happy to make any further changes or address any further issues.

Thank you for your consideration,

HEH, AQF, RNMA, MSA, and WFP

27 June 2020

Reviewer 1

This is an interesting manuscript that is very applied in focus, testing the effectiveness of the Gittings lionfish trap. It is a generally well written and concise manuscript that provides much useful information in thinking about approaches to managing lionfish populations in areas that are beyond the depth range of recreations SCUBA diving. I have a few broader comments and some suggestions for improvement, but do not think there is much work to be done for this to be in an acceptable form for publication.

Broader comments:

Methods:
I think we need a little more info in the methods about the artificial reefs. For example, how large are each of these structures. What’s the dominant habitat type that has established on them. What’s their relief relative to the seabed. What is the composition of the seabed around the structures that the lionfish will have to swim over to get to the traps. What’s the general water visibility typically like there - i.e. are traps in visual distance for fish from the structure. Does the point count for fish done by the diver in a 15m wide cylinder fully encompass the whole of each artificial reef?

The methods were clarified with the following text.

More information on the artificial reefs are provided in lines 125-134: “Gittings traps were deployed in depths of 33–37 m near eight artificial reefs on the nGOM Florida shelf. These included four poultry transport units (i.e., chicken coops), one steel pyramid, one cement mixer, and two military tanks located approximately 30 km south of Destin, Florida (**Fig. 3**). The artificial reefs were approximately 3 m x 2 m x 2 m (length x width x height) in size and deployed on sand bottom. No other known reef habitats (artificial or natural) were within at least 300 m of a given artificial reef. Lionfish density at each reef was surveyed immediately prior to trap deployment and trap retrieval. Surveys were conducted with point-counts by divers on SCUBA within a 15-m wide cylinder with the artificial reef at the center [46]. The diver survey included a lionfish count on the opposite sides of the artificial reef, followed by a count of lionfish within the reef structure [46,47].”

A sentence on underwater visibility was added in lines 148-151: “Underwater visibility ranged from 5–12 m, thus near traps were within direct visual range of the reefs by divers, intermediate distanced traps were sometimes within visual range or just outside, and far traps were outside of visual range.”

Details about the point count survey by divers were clarified: (lines 130-135) “Lionfish density at each reef was surveyed immediately prior to trap deployment and trap retrieval. Surveys were conducted with point-counts by divers on SCUBA within a 15-m wide cylinder with the artificial reef at the center [46]. The diver survey included a lionfish count on the opposite sides of the artificial reef, followed by a count of lionfish within the reef structure [46,47]. Lionfish density (fish per 100 m^2^) was computed as abundance (number counted) divided by the area sampled (177 m^2^).”

L121 and L126 - need to clarify why these n numbers are different. Also define what ‘paired’ means - are these two traps deployed near each other at the same distance from the artificial reef with the same soak time? What was the distance between paired traps. Could be worth adding a image of pairs traps to Figure 1 or Figure 3.

The methods now better explain the difference between the number of traps deployed (n = 82, used for calculated trap deployment success) and the number of trap configuration replicates (n = 58). As suggested below, a new table was added to show the trap configuration replicates. Single- vs. paired-treatments are also clarified. Updated text (lines 139-143): “Single-trap treatments included one trap deployed at the reef. Paired-trap treatments included two traps deployed simultaneously ~3 m apart. Deployment success (i.e., a trap landed upright and opened) for each trap deployment (n = 82 total traps deployed including paired treatments) was noted by SCUBA divers during the survey and unsuccessful deployments were corrected.”

We agree an image would be useful but, unfortunately, we do not have a good image of a paired trap deployment. Although we have GoPro videos from the divers, we do not have any high-quality images of side-by-side traps.

The breakdown of number of replicates between each of the factors is unclear (i.e. how many traps for each combination of number of traps, distance to reef, and soak time. Please add a summary table here that shows the number of replicates for each combination.

A summary table indicating the number of replicates by factor level (Table 2) was added it to the methods section (lines 157-159): “**Table 2. Replicates by deployment factor tested.** Number of Gittings trap replicates by deployment configuration with factors of soak time (0.25, 1, 4-5, 8, or 12-14 days), number of traps deployed (single or paired), and distance to artificial reef (5, 15, or 65 m).”

Please add a lot more detail on where the spearfishing data came from. Is this a scientific monitoring/lionfish culling dataset? Or does it come from recreation divers and lionfish derbies? Please elaborate on how fishers were able to capture all lionfish regardless of size - note the papers that suggest visual survey methods for lionfish/spearfishing are likely to be biased towards larger individuals anyway: e.g. [https://doi.org/10.1007/s00338-012-0987-8](https://urldefense.proofpoint.com/v2/url?u=https-3A__doi.org_10.1007_s00338-2D012-2D0987-2D8&d=DwQGaQ&c=sJ6xIWYx-zLMB3EPkvcnVg&r=LjBqIY5l82GH5E6-mpwPVjOnMm5D7qUkiVy95DypDBk&m=yVDVKnM8hYTke3vjoR85EVvJWbMu47Ly-7vjRcdrwBQ&s=S7cf1qWTCf5KLX9DJFbvOtc470PixiCgAWFUZhynv6Y&e=)

The data come from monthly data monitoring. Details for sampling methods were added to the text: (lines 175-182) “Lionfish size samples via spearfishing were collected during monthly lionfish culls by trained volunteer divers [51]. Spearfishing collections were subset for lionfish captured from artificial reefs during the same time period as the trap testing (June–December 2018). During spearfishing, divers attempted to capture all lionfish observed on a given artificial reef regardless of fish size. Lionfish detectability was assumed to be unbiased for larger individuals given the low structural complexity of the artificial reefs and the fact that previous serial removals on similar habitats produced homogeneous size distributions [46].”

Although lionfish detection in highly complex coral reefs are biased towards larger individuals (Greene et al 2012), results from lionfish detection studies on low complexity artificial reefs in the region where this study was conducted indicated that there was no size bias found during serial removals, i.e., results from repeated removals on the same reefs did not indicate smaller individuals were missed more often than larger ones (Harris et al. 2019).

Green SJ, Akins JL, Maljković A, Côté IM. Invasive lionfish drive Atlantic coral reef fish declines. Goldstien SJ, editor. PLoS One. 2012;7: e32596. doi:10.1371/journal.pone.0032596

Harris HE, Patterson III WF, Ahrens RNM, Allen MS. Detection and removal efficiency of invasive lionfish in the northern Gulf of Mexico. Fish Res. 2019;213: 22–32. doi:10.1016/j.fishres.2019.01.002

Also, there is evidence of recruitment peaks in lionfish populations. Was the spearfishing data collected over a similar time period to the trap data? Note, there is seasonality in size distribution of lionfish in some regions which may also affect the comparability of this data if not collected at the same time of year. e.g. [https://doi.org/10.7717/peerj.2730](https://urldefense.proofpoint.com/v2/url?u=https-3A__doi.org_10.7717_peerj.2730&d=DwQGaQ&c=sJ6xIWYx-zLMB3EPkvcnVg&r=LjBqIY5l82GH5E6-mpwPVjOnMm5D7qUkiVy95DypDBk&m=yVDVKnM8hYTke3vjoR85EVvJWbMu47Ly-7vjRcdrwBQ&s=QeSwOis5NmS3lhlys3ZwhvffMrm0AoOW2swRV4vvCFo&e=) If this is a problem, I think the comparisons between collection methods can still be included, but would note this issue in the discussion.

Yes, data from spearfishing were subset to only include lionfish speared on artificial reefs during the same time period as trap testing. This was stated in the Fig. 6 caption text, but now is more clearly indicated in the main text as well: (lines 177-178) “Spearfishing collections were subset for lionfish captured from artificial reefs during the same time period as the trap testing (June–December 2018).”.

Results:
Table 2 - Why is a soak time of 1 day set as the intercept? This confused me when I first looked at it, as the other other variables all use the smallest unit (i.e. single trap, 5 m distance from reef) as the intercept to compare others levels of those factors to. I suggest using the 3-6 hours soak time as the intercept to compare the 1 day, 4-5 days, and 12-14 days soak times to.

By default, R categorized the levels alphabetically and “1 d” was before “3-6 h”. As the reviewer suggested, we re-made models with the shortest time (3-6 hours were renamed to 0.25 days) used as the model intercept for the recruitment model, which we agree is easier to understand. The GLMM output (Table 3) was updated accordingly. Note that this does not statistically change the results, i.e., does not change z- nor p-values.

I know this is not directly a question you set out to address, so please feel free to ignore. However, it would be very interesting to know whether there is a relationship between size of lionfish caught in the trap and distance from reef the trap was. There’s a lot of literature on ontogenetic fish movements that would suggest that traps further from the reef would be more likely to be biased towards larger lionfish. Maybe add as a second panel to Fig 6?

This is an interesting idea. We tested if traps caught larger lionfish using a similar approach to comparing total length of lionfish caught by spear or traps, as suggested by the reviewer. The new results are presented in the study and a second panel is added to Fig. 6.

The results from this were as follows: (lines 281-282) “Lionfish sizes (measured in TL) were not significantly different between lionfish captured from traps deployed at distances 5 m and 15 m (**Fig. 6A,** KS test, P = 0.591)” . . .

Lines 291-297: **Fig 6. Lionfish size distributions by distance to reef and harvest gear.** A) Lionfish total length (TL) probability density distributions for lionfish captured in Gittings traps for traps deployed at near (5 m) and intermediate (15 m) distances to the adjacent artificial reef. B) Lionfish TL distributions for lionfish harvested by Gittings traps (all treatments) and lionfish sampled concurrently (June–December 2018) by spearfishing on similar northern Gulf of Mexico artificial reefs. Mean TL per distribution is indicated by a vertical dashed line.

Discussion:
There’s some numbers on bycatch from studies of lobster traps for lionfish - e.g. Pitt and Trott 2015, though the authors may be aware of other studies as well. It would be good to place the report numbers in L250 in the context of these other studies.

Pitt JM, Trott TM (2015) Trapping lionfish in Bermuda, part II: lessons learned to date. In: Proceedings of the 67th Gulf and Caribbean Fisheries Institute, Christ Church, Barbados, 3–7 November 2014, pp 221–224

Several sentences were added to summarize the work from Bermuda for the potential use of traps on their mesophotic reefs. This work has largely been disseminated in the GCFI Proceedings rather than peer-reviewed journals and we agree their work provides good context for the results of this study. I emailed Joanne Pitt (BIOS) this text (as well as the rest of it for context) who confirmed this was an accurate characterization of their work.

Lines 348-355: “Overall lionfish recruitment numbers to the Gittings trap were about double the numbers observed on lobster traps deployed near mesophotic reefs in Bermuda [34]. Subsequent experimental tests on Bermudan reefs with modified lobster trap funnels found that, similar to this study, lionfish catches were also highly variable and right-skewed with mean recruitment to the lobster trap structure ~3 lionfish per trap and ranging to >15 lionfish per trap [79]. While this variation was likely driven by the spatial variation observed in lionfish distributions on Bermuda reefs [9,80], our results unexpectedly indicated that lionfish density on an adjacent artificial reef was not a significant predictor of lionfish recruitment or catch by traps.”

General comments:

Fig 2 - Add scale bars and north arrows to all parts of the figure. The boundaries of the green study area box in the lower right panel seems to be positioned further south than the area indicated in the upper panel. I would suggest labeling each panel A, B, C and then providing a brief description of each panel in the legend.

Scale bars, north arrows, and letter labels were added to all three panels. The green study area box was corrected.

This manuscript makes extensive references to technical reports and videos. I think the nature of this study requires this, however, for long-term access web links to government sites etc are unlikely to be stable. I notice many of them are by Gittings who is a coauthor. Please could these technical reports and videos be cited via a DOI to ensure they are accessible to readers longer term?

A DOI was created to the Gittings et al. 2017 technical report:

Gittings SR, Fogg AQ, Frank S, Hart J V, Clark A, Clark B, et al. Going deep for lionfish: designs for two new traps for capturing lionfish in deep water. Silver Spring, MD; 2017. doi:10.13140/RG.2.2.22898.50886

Johnston et al. (2015) is hosted in the NOAA institutional repository, available <https://repository.library.noaa.gov/view/noaa/13954>.

The YouTube links are owned by the authors and will remain stable. We were unable to find a way to make a DOI for an online video.

Minor comments:

L18 - add by SCUBA - so that it reads: Spearfishing by SCUBA is currently…

Done.

L25 - be clearer what is meant by regulated species

Done. Now reads, “. . . four of which were species protected with regulations . . .”.

L49-50 - add word ‘invasive’ so that it reads: However, invasive lionfish have been…

Done.

L52 - mesophotic is defined from 40 - 200 m depth. So edit sentence to say: ‘lies within mesophotic and upper-bathyal depths of 40-300 m’

Done.

L53 - also see this recent review of mesophotic and upper-bathyal lionfish and their potential impacts in the Western Atlantic that may be useful reference to cite here:
[https://doi.org/10.1007/978-3-319-92735-0_48](https://urldefense.proofpoint.com/v2/url?u=https-3A__doi.org_10.1007_978-2D3-2D319-2D92735-2D0-5F48&d=DwQGaQ&c=sJ6xIWYx-zLMB3EPkvcnVg&r=LjBqIY5l82GH5E6-mpwPVjOnMm5D7qUkiVy95DypDBk&m=yVDVKnM8hYTke3vjoR85EVvJWbMu47Ly-7vjRcdrwBQ&s=Jn54YQz2f8yiI0Lsn4i-fGV7oNfwJ73U85hKj7O3lx4&e=)

Done. Cited.

L71 - change to: ‘However, it is critical’…

Done.

L96 onward - add some labels to Fig 1 to point out the key elements that are being described in the text. e.g. the trap jaws, the pivot axel, the netting, the lattice etc

Done. Key elements are now labelled on figure 1 (line 89) and figure 2 (line 124).

L111 - show location of Destin Fig 2

Done.

L130 - can you be more clear here that you are defining a variable called ‘Lionfish catch’ that you will be reporting on from the models. And also be more clear about defining “lionfish recruitment’ it wasn’t clear to me on my first read that you were defining terms here, and then I was confused when I got to the results how these terms were defined.

The text now states (lines 160-162), “Generalized linear mixed models (GLMMs) were computed to test the effect of deployment factors (i.e., number of traps, distance to artificial reef, and soak time) on 1) lionfish recruitment, 2) lionfish catch, 3) native reef fish recruitment, and 4) native reef fish (**Eq. 1**).”

These variables are made explicit in the equation notation in Eq. 1 (line 169):

Recruitment is now defined again for clarity in the results section: (line 214-215) “A total of 327 lionfish recruited (i.e., number of fish observed within the trap footprint) to Gittings traps . . . ”

L133 - ‘adjacent reefs’ - why is this plural? You mean the lionfish density on the single artificial reef next to the trap? Please rework this sentence to improve clarity

Yes. Clarified with the following text (line 163): “Lionfish density on artificial reef site was included as a covariate.”

L176-177 - This sentence on trap bycatch is very confusing. I know you explain what you mean in later sentences with the undetected individual, but I would remind readers upfront that ‘recruiting’ means diver in water observations and then give that result, and then remind readers that ‘’caught’ means identified on the surface in the trap after the dive and give the caught result. Then state that clearly there was a detection error.

Recruitment is now defined again for clarity in the results section and the text was clarified as follows (lines 221-226): “A total of 327 lionfish recruited (i.e., number of fish observed within the trap footprint) to Gittings traps during 82 trap sets (n = 58 deployments including paired sets) with 141 lionfish caught (**Fig. 4, left**). Trap bycatch of native species consisted of 28 individuals recruiting to the traps and 29 individuals caught from nine different species (**Fig. 4, right**). This result, where one more native species reef fish was caught (i.e., landed on the boat from the trap pull) versus documented as recruited, indicates an apparent detection error by SCUBA divers.”

L224 - these numbers are per trap right, not in sum for the pair? Please clarify

Yes. Updated text (line 243-244): “Mean lionfish recruitment to paired traps was 44% lower per trap compared to single traps . . .”. Throughout the text we “per trap” was added to clarify this.

L267 - I think you need to discuss that these results represent diver collected traps. So the intention is that these can longer-term be remotely deployed and recovered from the surface by boat, so need to trial in these conditions/verify that these results hold up with a boat recovery.

Updated text (lines 331-333): “As wide-scale commercial application for Gittings traps would be remote deployment and recovery from the surface by boat, future trials will be necessary to verify that these results remain consistent with these methods.”

The following text is also in the discussion (lines 379-382): “Similar gear testing to that conducted in this study will be needed for traps near natural reefs to evaluate their potential for habitat damage or bycatch, considering there are differences in benthic structure and community composition [14].”

L274 - also weight is a big consideration when deploying to much deeper reefs, i.e. winch for boats etc for recovery. As you say in your intro, there is a need for a method that could allow capture down to >300 m!

Agreed. Updated text (lines 309-314): “Trap mass will also be a consideration for commercial application. The traps in this study were built with relatively thick #6 rebar and a trap had a mass of ~35 kg, which made it difficult for a single person to move or deploy them. Moreover, the cost to bend this thick of rebar into the semi-circular jaw frames constituted >50% of their construction costs. Either #4 rebar (13 mm / 0.5 in diameter) or #5 rebar (15.875 mm / 0.625” diameter), which are both more easily sourced and manipulated, may provide better materials for trap jaws.”

L288 - though trapping removes worries around health and safety of divers in the water, and can have multiple simultaneous traps deployed

Agreed. A sentence was added in the introduction: (lines 64-65) “. . . Such technologies may offer a safe method for lionfish removal from deepwater reefs inaccessible to spearfishers and could allow a single vessel to multiply effort via simultaneous gear deployments.”

L292 - See the work of Green et al 2014 that specifically identifies target thresholds for culling to translate to ecological benefits. There is a need to calculate these thresholds for mesophotic reefs, and then consider whether traps can reach them: [https://doi.org/10.1890/13-0979.1](https://urldefense.proofpoint.com/v2/url?u=https-3A__doi.org_10.1890_13-2D0979.1&d=DwQGaQ&c=sJ6xIWYx-zLMB3EPkvcnVg&r=LjBqIY5l82GH5E6-mpwPVjOnMm5D7qUkiVy95DypDBk&m=yVDVKnM8hYTke3vjoR85EVvJWbMu47Ly-7vjRcdrwBQ&s=CEH1LnJeQ_Lf_lJQZAb1avJlOjg-f-E8auiuBcE0oJI&e=)

We agree, but would contend that ecosystem models may be necessary to model the complexity and nonlinear dynamics for considering density dependence. The bioenergetic model in Green et al. 2014 predicted an 88% reduction in lionfish density on Caribbean patch reefs was required to achieve a 75% increase in lionfish prey biomass, indicating that partial removals could mitigate lionfish effects on native prey fish. However, manipulation of lionfish density on Caribbean patch reefs showed lionfish have density-dependent predation rates and native fish biomass did not increase if lionfish density was above a threshold value of 5 fish per 100 m^2^ (Benkwitt 2015). Density-dependent processes may explain results of studies from throughout the invaded range that show sustained lionfish removal efforts had no discernable positive impacts on native communities. Dahl et al. (2016) found no detectable increase in small demersal reef fish density or species diversity during a two-year lionfish removal experiment on nGOM artificial reefs. Smith et al. (2017) reported no increase in native reef fish biomass after 21 months of lionfish removals on coral patch reefs in The Bahamas. Lionfish removal treatments conducted every month or four-months in Biscayne National Park in SE Florida did not change the biomass of lionfish nor biomass of native predators compared to control reefs (C. Stallings, unpublished), and Harms-Tuohy et al. (2018) reported no increase in native reef fish densities from lionfish removals on ~15,000 m^2^ of continuous coral reef in Puerto Rico. The failure to detect positive community-level results may be due to reef fish recruitment and succession dynamics operating on longer time scales than what could be captured in the studies. Alternatively, incomplete detection and removal of lionfish may have the left lionfish densities above threshold levels needed to foster community recovery, as suggested by Benkwitt (2015). The remaining lionfish would be released from density-dependent competition and cannibalism, thus these individuals would likely have higher predation rates and disproportionate impacts on the reef fish community.

Green, S.J., Dulvy, N.K., Brooks, A.M.L., Akins, J.L., Cooper, A.B., Miller, S., Coté, I.M., 2014. Linking removal targets to the ecological effects of invaders: a predictive model and field test. Ecol. Appl. 24, 1311–1322. <https://doi.org/10.1890/13-0979.1>

Dahl, K.A., Patterson, W.F., Snyder, R.A., 2016. Experimental assessment of lionfish removals to mitigate reef fish community shifts on northern Gulf of Mexico artificial reefs. Mar. Ecol. Prog. Ser. 558,

Smith, N.S., Green, S.J., Akins, J.L., Miller, S., Côté, I.M., 2017. Density-dependent colonization and natural disturbance limit the effectiveness of invasive lionfish culling efforts. Biol. Invasions 19, 2385–2399. <https://doi.org/10.1007/s10530-017-1449-6> 207–221.

Harms-Tuohy, C.A., Appeldoorn, R.S., Craig, M.T., 2018. The effectiveness of small-scale lionfish removals as a management strategy: effort, impacts and the response of native prey and piscivores. Manag. Biol. Invasions. <https://doi.org/10.3391/mbi.2018.9.2.08>

Benkwitt, C.E., 2015. Non-linear effects of invasive lionfish density on native coral-reef fish communities. Biol. Invasions 17, 1383–1395. <https://doi.org/10.1007/s10530-014-0801-3>

At some places in the manuscript you refer to people who fish as ‘fishers’ in others you use ‘fishermen’. I would recommend standardizing to fishers, or be clearer why there’s a need for the two terms.

“Fishermen” on line 70 was changed to “fisher”. The term is not used elsewhere.

Reviewer 2

Overall the manuscript was clearly and concisely written and provides valuable information about an important marine invasive species of concern throughout the Western Atlantic. The research of the efficacy of utilizing a non-containment trapping device to capture lionfish near artificial reefs is important for improving management strategies regionally to control lionfish populations on mesophotic reefs. The paper highlights the potential of these traps to capture lionfish and a produces a capture rate comparable to trapping of other native target species using fish traps. It discusses recommendations for future directions of research to help improve upon this potential methodology by reducing escape rates of lionfish and placing traps at optimal distance to reefs.
The manuscript is recommended for acceptance with minor revisions described below.

Many of the reviewer comments or requests for clarifications overlapped with those of Reviewer 1, indicating a clear need for better description or clarity in a few areas. Addressing these improved the manuscript.

Major Issues
1. The author should be careful to not overstate findings when discussing the significance of larger size of lionfish in traps. There are a few causes of this result that were not addressed and should be if possible (month(s) which most lionfish were capture from traps compared to spear, is there size estimates of all recruits from diver surveys that can be looked at to see if only larger fish recruited to traps. Did more small fish escape the traps.

We tried to walk back the strength of our conclusions, consider alternatives, and more clearly state data limitations where they exist. In the discussion we hypothesize possible selectivity for larger fish and what the potential implications could be for a commercial trap fishery. We also clarified that spearfishing samples were conducted concurrently, as described to addressing reviewer 1’s comments.

The discussion text was updated as follows (lines 359-368): “A possible selectivity demonstrated by Gittings traps for larger lionfish could have implications for depleting their populations. Smaller lionfish may have escaped from the traps at greater rates and, in retrospect, it would have been useful to collect size estimates of the lionfish by divers prior to trap retrieval. Alternatively, traps may attract larger individuals. Higher movement rates for larger lionfish is reasonable as they are more physically capable to make such movements and face lower risk of predation in transit [85,86]. Juvenile lionfish risk conspecific predation [82,87], particularly in areas of high lionfish density [83] where this study was conducted, and smaller individuals may thus occupy smaller foraging arenas near reefs [88]. Given fecundity increases exponentially with length [89–91], a gear selectivity bias toward larger fish may have a greater impact on reducing lionfish egg production.”

2. The author fails to discuss the fact that no native fish escaped from the trap. This is relevant information since upwards of 50% of lionfish escaped. Authors should provide a better description of the native fish behavior observed in the traps during this study which could help with improvement to future design.

The following text was added/updated (lines 213-219): “. . . lionfish escapement was indicated with 56% of the lionfish that had recruited to traps escaping during retrieval. The proportion of lionfish that escaped did not appear to correlate with higher recruitment of lionfish to traps (see R code for scatter plots). Because traps closed during ascent when SCUBA divers filled lift bags attached to traps with air, trap closing took approximately 3–5 seconds and divers often observed lionfish swimming out in this time. Native species were not observed escaping during trap closing; however, detection of native fishes was an apparent issue.”

3. Authors failed to describe what happened to the native fish that were captured in the traps? Were they descended and was that successful (ie did the bycatch survive)? Did the authors estimate the size of these fish? Were they on par with the sizes of lionfish in the traps?

Native species bycatch were retained for measurement and life history workups. The following text was added (lines 231-232): “Native species TL measurements were similar to lionfish with a range of 151–350 mm.”

4. The author should provide more detailed information about the density of the lionfish populations at each site. Without this information it will be difficult for future studies assessing efficacy of capture methods to compare their results to this current study. Will these traps work at all on sites with much lower lionfish densities?

Agreed. The following text was added (lines 250): “Lionfish density on the artificial reef study sites ranged from 6 to 36 fish per 100 m².”

The discussion ends with us considering the potential use of Gittings traps to target lionfish on natural reefs and precisely the critical question that these traps be may not be effective at attracting and capturing lionfish on sites with much lower lionfish densities, which would prevent them from being economically viable. Updated text: (lines 381-390) “It is also unknown whether lionfish attraction will be higher for traps near natural versus artificial reefs. Lionfish densities on nGOM artificial reefs are >10X higher than on nGOM natural reefs [3,46,55], which suggests a trap structure may readily attract lionfish from natural reefs. However, if lionfish movement and home range are largely driven by density-dependence or food availability [14,61,84], then the substantially lower densities on natural reefs may critically reduce catch rates. The potential economic viability of a lionfish trap fishery should be carefully considered. Future trap field testing could examine design strategies to increase lionfish attraction (e.g., using lights, sound, or different structures) and technoeconomic assessments may identify capital and operational expenses to evaluate what catch rates could make lionfish trapping commercially feasible.”

Minor issues
Page: 8
Line 25: Is this species or individual fish

Number of fish. Clarified as follows: (lines 25-26) “. . . 28 fish that were native species (four were species protected with regulations) . . .”

Line 25: which "are" regulated

Done.

Line 26: Here you have 29 fish captured which is higher than the 28 recruited unless the former is species not individual fish. These sentences are a bit confusing in the abstract. More clearly explained in results.

Updated text (lines 27-28): “. . . catching 144 lionfish and 29 native species (1 more than recruited indicating detection error).” We also try to clarify the confusion caused by this in the results.

Line 29: Is this captured or recruited? Can you get estimates on recruited from videos? Would be interesting to know if the smaller fish are more likely to escape or just less likely to recruit to the trap structure. Need to set up some stereocameras probably to get at that question.

As shown in the photos and Figure 2, we attempted to use time-lapse cameras to document higher resolution recruitment to the traps. The following text was added to briefly explain this attempt and inability to use camera data (lines 154-156): “Although we attempted to use time-lapse camera units to document higher resolution recruitment to the traps, the camera failure rates during this study were too high for the data to be useful.”

Page 9
Line 48 "is a top"

Done.

Line 53 - This may be an overstatement at the knowledge of population densities at mesophotic depths throughout the western Atlantic since site specific information is very limited to date.

I understand the sentiment that we know relatively little about mesophotic reef systems. However, authors of four studies have concluded lionfish densities on mesophotic reefs are higher than on shallower depths less than 40 m. The text has been updated as follows (lines 56-57): “Although survey capacity for deepwater reefs are relatively limited [25], lionfish densities are often higher than on corresponding shallower reefs [9,26–28].”

Page 10
Line 66: Should this be Figure as well if you are introducing a descriptive table, for a ready that has not seen the trap a figure would have more use at this time?

Done. Figure 1 is now introduced here rather than several paragraphs later in the methods. Key design elements are also now labeled, as suggested by reviewer 1.

Line 71: "economic viability" and potential undesirable effects.

Done.

Page 11
Line 85 - This is different that what was reported in abstract. Compared to spearfishing catch

The text was updated to fix this (lines 96-97): “Lionfish size distributions was compared between Gittings trap catches by distance to the artificial reef and *in situ* size distributions obtained from spearfishing catches.”

Page 12
Line 110 - Four of these sites look quite close together compared to the other sites. Did you account for distance to next structure when analyzing recruitment or capture results or lionfish densities at site?

The minimum distance between sites was 300 m, which is now explicit in the text. To our knowledge, there were no other structures within this radius. We did not account for proximity to other sites in the analyses. This would be interesting, but our goal was to look at many different factors for deployment strategies, which limited the number of factors we could test statistically.

The text was updated to clarify this information: (lines 125-130) “Gittings traps were deployed in depths of 33–37 m near eight artificial reefs on the nGOM Florida shelf. These included four poultry transport units (i.e., chicken coops), one steel pyramid, one cement mixer, and two military tanks located approximately 30 km south of Destin, Florida (**Fig. 3**). The artificial reefs were approximately 3 m x 2 m x 2 m (length x width x height) in size and deployed on sand bottom. No other known reef habitats (artificial or natural) were within at least 300 m of a given artificial reef.”

Did you look at artificial reef type when analyzing recruitment results or lionfish densities at site?

This would be interesting, considering the plans and discussion for testing how these traps will be able to attract lionfish from natural reefs, but our sampling lacks the power to incorporate more factors into the analysis, particularly given the unbalanced design with different artificial reef types. Reef type was not included as a fixed effect, but individual reef site was included as a random effect in the GLMM. Specifically, we were interested in estimating the mean-differences by different deployment configurations (i.e., soak time, distance from reef, and number of traps deployed), and less interested in knowing the different means by reef type. Thus, we included reef site as a random effect in the model to account for repeated measures and the overall variation attributed by individual reefs and that were sampled multiple times with different deployment configurations and soak times. Background information for our specification of fixed or random effects is provided in Bolker at al. 2009, <https://doi.org/10.1016/j.tree.2008.10.008>.

Line 116 - Did you estimate size on these surveys as the in situ size estimates referred to above?

Unfortunately, no. This would have been prudent. We now state this error in our experimental design in hopes that in future research will avoid our mistake. Updated text (lines 360-362): “Smaller lionfish may have escaped from the traps greater rates and, in retrospect, it would have been useful to collect size estimates of the lionfish by divers prior to trap retrieval.”

Page 13
Line 127 - Need to explain why traps were retrieved by divers and not set as a surface buoy. This would not be a practical way to deploy and retrieve these traps commercially.

This is true, and we agree that diver retrieval is not a practical commercial application for traps. However, it was not our intention to test traps for commercial use. Significant more R&D will be necessary for that. Rather, our objective tests to examine if the traps worked to recruit lionfish and guide future research and development: lines (100-102) “We consider how findings from this initial study can inform further research and development of lionfish traps and innovative harvest technologies to control deepwater lionfish populations.”.

Text in the discussion was updated consider commercial applications: (lines 301-309) “Given that wide-scale commercial use of Gittings traps would entail remote deployment and recovery from the surface, design iterations and field tests will be necessary to improve deployment success and lionfish catch rates. Potential gear modifications could include the following: adjustments to flotation and ballast that could help traps keep a vertical orientation during descent to allow successful opening, a reconfigured harness that closes the jaws more quickly and keeps them closed during recovery, a looser net that could allow more billowing and not contact lionfish during closure, and faster trap retrieval provided by a shipboard winch.”

Line 130 - Was there a size estimate conducted here by the diver? Line 135 - Same comment as above...

Unfortunately, size estimates were not taken by divers. We make this explicit: (lines 360-362) “Smaller lionfish may have escaped from the traps at greater rates and, in retrospect, it would have been useful to collect size estimates of the lionfish by divers prior to trap retrieval.”.

Four of these sites look quite close together compared to the other sites. Did you account for distance to next structure when analyzing recruitment or capture results or lionfish densitiesor at site?

The minimum distance between reefs was 300 m. To our knowledge, there were no additional structures between reefs where the habitat is just open sandy bottom areas. Clarification in the text is described above.

No, we did not account for distance in the analysis. We have the data to do so but lack statistical power to include it in the GLMMs.

Did you look at artificial reef type when analyzing recruitment results or lionfish densities at site?

Repeated question. See answer above.

Page 14
Line 145 - was this done at same time period as the trap study? Within a week/month/year? That could greatly influence size distribution.

As indicated by the other reviewer, it is important to clarify this information. The text was updated as follows (lines 175-182): “Lionfish size samples via spearfishing were collected during monthly lionfish culls by trained volunteer divers [51]. Spearfishing collections were subset for lionfish captured from artificial reefs during the same time period as the trap testing (June–December 2018). During spearfishing, divers attempted to capture all lionfish observed on a given artificial reef regardless of fish size. Lionfish detectability was assumed to be unbiased for larger individuals given the low structural complexity of the artificial reefs and the fact that previous serial removals on similar habitats produced homogeneous size distributions [46].”

Line 150
What did you do with the native species captured? Did you descend them? Assess condition upon release and mortality?

Native species bycatch were retained for measurement and life history workups. The following text was added (lines 227-228): “Native species TL measurements were similar to lionfish with a range of 151–350 mm.”

Line 152 - Was there any issues with trap entanglement with habitat?

No. The following text was added (lines 231-232): “Traps landed on sand and there were no entanglement issues with habitat.”

Page 15
Line 177 – Ok this is clear here but this detail makes the abstract a bit confusing without it being explained.

Updated abstract text (lines 28): “. . . 29 native species (one more than recruited indicating detection error).” As mentioned, we also clarify this in the results and have tried to improve this with the revisions.

Line 170 – 183 - I would reword this as "Four native species capture in the traps were regulated species:.... Then… "Addition native species catches consisted of 15 sand perch....

Done

Line 183 -There is no discussion at all about why all native fish that recruited to the traps were captured. Did any of them try to escape as it was being retrieved? This would be worth discussing to potentially brainstorm why lionfish are more likely to escape and what modifications can be made to take into account their different behavior. Did a high proportion of lionfish escape when their were more total fish in the trap before retrieval?

We made these scatter plots to consider this question. They indicate that while variation increased with higher recruitment there is no trend towards a higher proportion of escapement with higher recruitment.

Text was added and updated to consider the issue of lionfish versus native species escapement, detailed below (lines 213-219): “. . . lionfish escapement was indicated with 56% of the lionfish that had recruited to traps escaping during retrieval. The proportion of lionfish that escaped did not appear to correlate with higher recruitment of lionfish to traps (see R code for scatter plots). Because traps closed during ascent when SCUBA divers filled lift bags attached to traps with air, trap closing took approximately 3–5 seconds and divers often observed lionfish swimming out in this time. Native species were not observed escaping during trap closing; however, detection of native fishes was an apparent issue.”

Observations about native fishes were added: (lines 224-226): “This result, where one more native species reef fish was caught (i.e., landed on the boat from the trap pull) versus documented as recruited, indicates an apparent detection error by SCUBA divers.”

Potential gear modifications are considered in lines 304-309: “Potential gear modifications could include the following: adjustments to flotation and ballast that could help traps keep a vertical orientation during descent to allow successful opening, a reconfigured harness that closes the jaws more quickly and keeps them closed during recovery, a looser net that could allow more billowing and not contact lionfish during closure, and faster trap retrieval provided by a shipboard winch.

Page 16
Line 189 - remove "to"

Done

Line 192 - Since this influences catch would be interesting to look at effects of having multiple artificial reefs nearby.

The difference was significant between the 5 or 15 m and 65 m distances. We assume because the reefs were over 300 m apart and that this would likely have negligible effects given the apparent lack of recruitment to the traps deployed 65 m from the reef.

Line 193 - But did the two traps catch more lionfish in total than one? Because that would suggest it might still worth putting two traps down on a site, particular if they are connected and you can deploy and retrieve them with minimal additional effort than two.

The results show the variance was high and the difference in the means between the two was not significant. The p-value indicates that we fail to reject the null that the two levels did not significantly differ. The results appear to the indicate that the extra effort of deploying two traps per site is not worthwhile.

Line 199 - Did you have video data to see if lionfish had recruited to the traps within the 1-5 days when recruitment is highest but then left before you retrieved it day 12-14?

As discussed above, we attempted to use time-lapse cameras to document higher resolution recruitment to the traps. The following text was added (lines 154-156): “Although we attempted to use time-lapse camera units to document higher resolution recruitment to the traps, the camera failure rates during this study were too high for the data to be useful.

Line 202 - How low was the lowest density. Is this because density was just relatively high at all sites?

The following text was added (lines 250): “Lionfish density on the artificial reef study sites ranged from 6 to 36 fish per 100 m².”

Page 18
Line 235
As commented above. Were the fish speared during the same time period (within the same month at least) because if not this could explain less juveniles.

Agreed. Yes. Addressed above.

Did you look at videos or estimates during surveys to see if smaller fish escape more easily or are they less likely to recruit?

Unfortunately, the videos could not help discern this. See discussion of the time-lapse cameras above.

Page 19
Line 242 - I see the time period here. It would be worth checking if the portion of fish caught in the summer months in traps is similar to the fish caught in the summer months spearing. Since the trap catch sample size is relatively low (especially compared to spearing sample size); if a larger proportion of fish were caught in winter just this could explain less small fish.

Yes, we might expect seasonal changes in mean population size, but there is not enough resolution in the data to test this among months. Catches did not appear to be different among months during the time period of the study.

Line 251 - Change Would be expected to "was expected"

Done.

Line 256 – Is there any evidence of them leaving? Camera footage?

The following text was added (lines 216-219): “Because traps closed during ascent when SCUBA divers filled lift bags attached to traps with air, trap closing took approximately 3–5 seconds and divers often observed lionfish swimming out in this time. Native species were not observed escaping during trap closing; however, detection of native fishes was an apparent issue.”

Line 258 - But lionfish were still present during the long soak times no just slightly lower densities? This suggests that native fish may just take longer to recruit not that lionfish necessarily deter them. Unless you compared traps with and without lionfish with native predators densities, but I didn't see that in the results.

The text has been updated as follows (lines 342-347): “This may also suggest that lionfish recruit relatively quickly to traps but leave during longer soak times, while native fish may take longer to recruit to traps. Our observations of higher lionfish and lower native species recruitment during shorter soak times, and the converse for longer soak times, supports the idea that lionfish presence deters or excludes native reef fishes, which has been reported in other studies on the indirect effects of lionfish on native species [75–78].”

Page 20

Line 261 - As stated above, it would be good to know whether the total number of lionfish for the two traps was larger or similar to that of one trap and not just the per trap density. It seems from the table that it is likely the same.

Yes, they were similar. The difference in the means of per trap catch and recruitment were not significant (Table 3).

Line 263 - As state above in Results.
What type of densities are we comparing here? That would be useful for the reader to know to understand whether artificial reefs may be at carrying capacity or etc. Were they all relatively high density?

The following text was added (lines 250): “Lionfish density on the artificial reef study sites ranged from 6 to 36 fish per 100 m².”

Line 271 - Wouldn't this loose net increase risk of entangement and ghost fishing

Traps are placed on sand and low relief habitats where snagging was less likely. We do not think the extra net would significantly change the design and increase the risk of entanglement or ghost fishing.

Page 21
Line 297 - You don't know it is higher movement/recruitment of large individuals to the traps. Maybe the small fish escaped the trap more easily and did not get captured. Be careful not to overstate a result here about smaller fish movement.

We tried to walk-back the conclusions in the discussion: (lines 360-368): “Smaller lionfish may have escaped from the traps at greater rates and, in retrospect, it would have been useful to collect size estimates of the lionfish by divers prior to trap retrieval. Alternatively, traps may attract larger individuals. Higher movement rates for larger lionfish is reasonable as they are more physically capable to make such movements and face lower risk of predation in transit [85,86]. Juvenile lionfish risk conspecific predation [82,87], particularly in areas of high lionfish density [83] where this study was conducted, and smaller individuals may thus occupy smaller foraging arenas near reefs [88]. Given fecundity increases exponentially with length [89–91], a gear selectivity bias toward larger fish may have a greater impact on reducing lionfish egg production.”

Line 306 - This sentence (Harvest…) is confusing, not sure exactly what you are saying here

Restated as follows (lines 371-373): “Harvest of faster-growing juveniles [54,55,93] contributes to growth overfishing [94], and age-structured lionfish population models indicate controlling lionfish population growth requires removing smaller individuals [23,24].”

Page 22
Line 318 - But that is because there is no other structure for them to attract to which is not the case as natural reefs. I don't think we can assume traps will readily pull lionfish from natural reefs.

Agreed. The discussion ends considering this critical question and their potential use on natural reefs (lines 381-388): “It is also unknown whether lionfish attraction will be higher for traps near natural versus artificial reefs. Lionfish densities on nGOM artificial reefs are >10X higher than on nGOM natural reefs [3,46,55], which suggests a trap structure may readily attract lionfish from natural reefs. However, if lionfish movement and home range are largely driven by density-dependence or food availability [14,61,84], then the substantially lower densities on natural reefs may critically reduce catch rates. The potential economic viability of a lionfish trap fishery should be carefully considered. Future trap field testing could examine design strategies to increase lionfish attraction (e.g., using lights, sound, or different structures) . . .”

Figure 1C – what is that top line attached to in the schematic? Above the float?

The subsurface float. This and other key items on figures 1 and 2 are now labeled, as suggested.

Figure 6 – Density on the y axis is confusing here since it is used in earlier figures and tables to represent density of fish per trap

Agreed. The y-axis title has been changed to "Probability density".

*UPDATE 29 JUNE. Letter from the journal:.*

PONE-D-20-07219R1

Testing the efficacy of lionfish traps in the northern Gulf of Mexico Mr. Holden Earl Harris

Dear Mr. Harris,

Thank you for submitting your manuscript entitled "Testing the efficacy of lionfish traps in the northern Gulf of Mexico" to PLOS ONE. Your manuscript files have been checked in-house but before we can proceed we need you to address the following issues:

1. In your Methods section, please provide additional information regarding the permits you obtained for the work. Please ensure you have included the full name of the authority that approved the field site access and, if no permits were required, a brief statement explaining why.

The following text was added in the methods: (lines 189-185) “Spearfishers were informed and consented to information about their catch being used for research purposes. Lionfish collection by researchers followed humane sampling protocol with euthanasia via pithing the brain case as reviewed and approved by the University of Florida’s Institutional Animal Care and Use Committee (UF IACUC Protocol #201810225). Authorized use of Gittings traps in US federal waters to collect lionfish for scientific research was granted by a letter of acknowledgment from the US National Marine Fisheries Service Southeast Regional Office in accordance with the definitions and guidance at 50 CFR 600.10.”

Note that this text had previously been in our ethics declaration and additional information. The line numbers have been updated in this document.

2. Please remove your figures/ from within your manuscript file, leaving only the individual TIFF/EPS image files. These will be automatically included in the reviewer’s PDF.

Done. We had included low-resolution thumbnail images for ease of reference. These have been removed.
